# Integrated histopathological, lipidomic, and metabolomic profiles reveal mink is a useful animal model to mimic the pathogenicity of severe COVID-19 patients

**DOI:** 10.1038/s41392-022-00891-6

**Published:** 2022-01-28

**Authors:** Zhiqi Song, Linlin Bao, Wei Deng, Jiangning Liu, Erjun Ren, Qi Lv, Mingya Liu, Feifei Qi, Ting Chen, Ran Deng, Fengdi Li, Yunpeng Liu, Qiang Wei, Hong Gao, Pin Yu, Yunlin Han, Wenjie Zhao, Junjun Zheng, Xujian Liang, Fuhe Yang, Chuan Qin

**Affiliations:** 1 NHC Key Laboratory of Human Disease Comparative Medicine, Beijing Key Laboratory for Animal Models of Emerging and Remerging Infectious Diseases, Institute of Laboratory Animal Science, Chinese Academy of Medical Sciences and Comparative Medicine Center, Peking Union Medical College, Beijing, China; 2 Shi Jia Zhuang Academy Of Agricultural And Forestry Sciences, Shijiazhuang, China; 3 Institute of Special Animal and Plant Sciences of CAAS, Changchun, China; 4 Chinese Academy of Agricultural Sciences (CAAS), Beijing, China

**Keywords:** Infection, Infectious diseases

## Abstract

Severe acute respiratory syndrome coronavirus 2 (SARS-CoV-2) is transmitted on mink farms between minks and humans in many countries. However, the systemic pathological features of SARS-CoV-2-infected minks are mostly unknown. Here, we demonstrated that minks were largely permissive to SARS-CoV-2, characterized by severe and diffuse alveolar damage, and lasted at least 14 days post inoculation (dpi). We first reported that infected minks displayed multiple organ-system lesions accompanied by an increased inflammatory response and widespread viral distribution in the cardiovascular, hepatobiliary, urinary, endocrine, digestive, and immune systems. The viral protein partially co-localized with activated Mac-2^+^ macrophages throughout the body. Moreover, we first found that the alterations in lipids and metabolites were correlated with the histological lesions in infected minks, especially at 6 dpi, and were similar to that of patients with severe and fatal COVID-19. Particularly, altered metabolic pathways, abnormal digestion, and absorption of vitamins, lipids, cholesterol, steroids, amino acids, and proteins, consistent with hepatic dysfunction, highlight metabolic and immune dysregulation. Enriched kynurenine in infected minks contributed to significant activation of the kynurenine pathway and was related to macrophage activation. Melatonin, which has significant anti-inflammatory and immunomodulating effects, was significantly downregulated at 6 dpi and displayed potential as a targeted medicine. Our data first illustrate systematic analyses of infected minks to recapitulate those observations in severe and fetal COVID-19 patients, delineating a useful animal model to mimic SARS-CoV-2-induced systematic and severe pathophysiological features and provide a reliable tool for the development of effective and targeted treatment strategies, vaccine research, and potential biomarkers.

## Introduction

Severe acute respiratory syndrome coronavirus 2 (SARS-CoV-2) has caused devastating adverse effects on global health since 2019. Several experimental animals, such as nonhuman primates (*Macaca mulatta*),^1–3^ genetically modified mice,^4,5^ golden hamsters (*Mesocricetus auratus*),^6^ and ferrets (*Mustela putorius furo*)^7,8^ can be infected by SARS-CoV-2 in the laboratory. However, the infection of animals including pets and domestic, rural, agricultural, working, or zoological animals that are in close contact with humans is highly risky. SARS-CoV-2 infections have been reported in cats and dogs as pets, tigers, and lions in zoos, and both pet and wild ferrets. Unexpectedly, a farmed mink (*Neovison vison*) infected with SARS-CoV-2 was detected in the Netherlands on April 23, 2020, leading to a rapid and widespread outbreak in Denmark and other countries in June 2020.^9^ SARS-CoV-2 transmission is not only very easy between minks within a farm but can also spread to and from people with close contact to farmed minks.^10^ This virus was initially introduced by humans to minks and has since been transmitted between the two species.^11^ During the widespread circulation among minks and humans, virus variants that mutated in minks were also transmitted from minks to humans.^10,11^ It is apparent that minks are highly susceptible to SARS-CoV-2 infection, and two recent studies have reported that SARS-CoV-2 can efficiently replicate in the respiratory tract and effectively transmit via respiratory droplets in minks.^12^ Owing to the increased attention paid to the histopathological features of lung lesions, detailed and systemic histopathological findings from minks infected with SARS-CoV-2 are rare. While, a useful animal model that mimics severe and fatal COVID-19 patients in terms of the induced multiply organs lesions, systemic metabolic disorders, and pathogenesis is urgently needed to improve the screening and exploration of effective and targeted treatment strategies, vaccine research, and potential biomarkers.

Here, minks were challenged with SARS-CoV-2 and observed for 14 days, and the systemic histopathological findings and metabolic characteristics of the infected minks during the duration were first recorded. Exploring the pathological and metabolic features not only contributes to understanding the pathophysiologic characterization of SARS-CoV-2-induced systemic disease in minks but also provides a potential opportunity to develop the mink as an effective animal model of SARS-CoV-2 infection to explore prevention and treatment.

## Results

### Clinical features, viral RNA distribution, and severe histological lesions in the respiratory system of minks infected with SARS-CoV-2

To investigate viral replication and histopathological changes, 18 minks were inoculated with 1 × 10^6^ 50% tissue-culture infectious dose (TCID_50_) per mL of SARS-CoV-2 stock virus via the intratracheal route. There were no significant changes in body weight or temperature (Supplementary Fig. 1a, b) in the infected minks compared with the negative-control minks, except for a transient decrease by 76 g (7.5%) of the body weight at 1-day post infection (dpi) in one mink which was euthanized. One mink displayed moderate respiratory signs; the clinical score reached a peak value of 2 and lasted for 4 days at 8–11 dpi during the monitoring duration of 14 days (Supplementary Fig. 1c and Supplementary Table 1). Next, we determined the viral RNA loads in nasal lavage fluids, throat swabs, and anal swabs. The viral loads from the nasal lavage fluids and throat swabs both lasted for 10 days, peaked at 1–2 dpi (7.15 log_10_ RNA copies/mL and 6.73 log_10_ RNA copies/mL), and then dropped to undetectable levels at 12 dpi (Supplementary Fig. 1d, e). The viral loads from anal swabs lasted for 8 days and peaked at 6 dpi (3.65 log_10_ RNA copies/mL) (Supplementary Fig. 1f). After euthanization at different timepoints, viral RNA was detected by quantitative reverse-transcription polymerase chain reaction (qRT-PCR) in different lobes of the lungs and the brain, eyeball, and ovary to investigate the dissemination of SARS-CoV-2 in minks. Infection of minks with SARS-CoV-2 resulted in a high mean viral RNA load in the lung tissues (5.02 log_10_ RNA copies/mL). At 6 dpi, the collected brains and eyeballs from two infected minks had detectable viral RNA, with mean viral RNA loads of 3.34 log_10_ RNA copies/mL and 3.56 log_10_ RNA copies/mL, respectively (Supplementary Fig. 1g). Our previous research demonstrated that rhesus macaques could be infected with SARS-CoV-2 via the ocular conjunctival route and develop mild COVID-19.^13^ In contrast, some severe patients with COVID-19 have ocular manifestations such as chemosis and conjunctival congestion.^14^ Our findings provide laboratory evidence of this phenomenon.

**Fig. 1:**
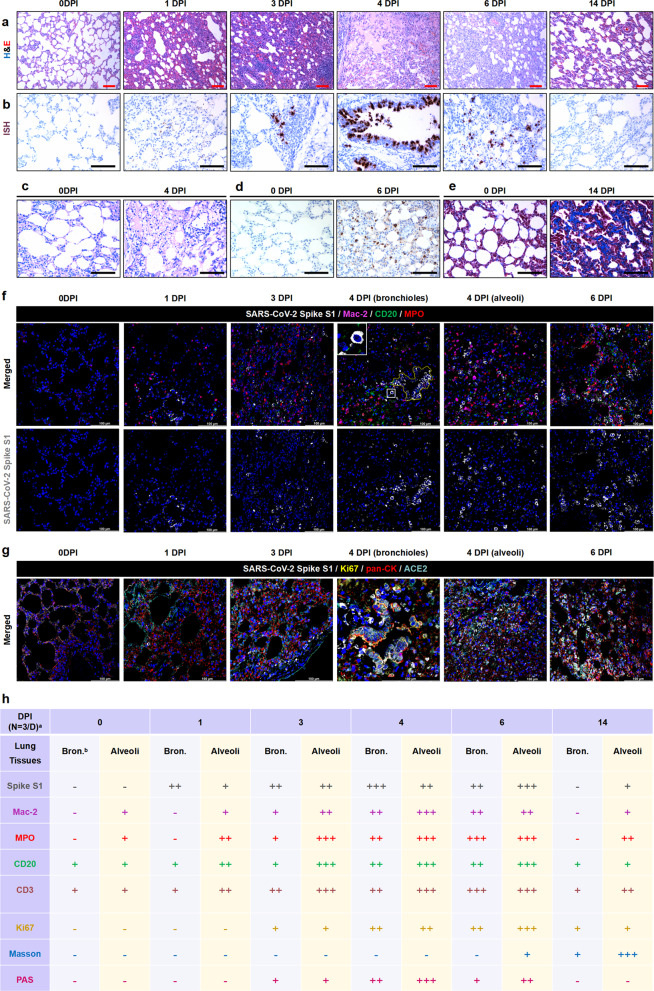
Histopathological feature of the lungs in infected minks during the duration of monitoring (*n* = 3/group). **a** The histopathological lesions in the lungs were observed by hematoxylin and eosin staining. **b** Viral RNA was examined by in situ hybridization. **c** There was more exudate stained by periodic acid-Schiff staining at 4 dpi compared with other timepoints. **d** There was more viral S protein in the alveoli detected by immunohistochemistry at 6 dpi. **e** Aggregation of collagenous fibers at 14 dpi was stained by modified Masson staining. **f** The relationship of viral antigen (S1 protein; gray) and the infiltration of inflammatory cells including Mac-2^+^-activated macrophages (magenta), CD20^+^ B lymphocytes (green), and MPO^+^ neutrophils (red) was analyzed by multiplexed immunohistochemistry. Viral S protein was distributed in the bronchioles (yellow line) at 4 dpi (L). **g** The distribution of viral antigen (gray), proliferative cells (yellow), pulmonary epithelial cells (red), and ACE2 receptor (cyan) were examined by mIHC. **h** Summary and comparison of the viral antigen and immune cell distributions as well as the histopathological features of lungs in the infected minks. ^a^Three minks were observed and calculated in each group. ^b^The bronchiole (bron.) and alveoli were observed and recorded in each mink. “−”, none. “+”, a few. “++”, some. “+++”, abundant. Data are representative of three independent experiments. Red scale bar at 100× = 100 µm, black scale bar at 200× = 100 µm. The white scale bar at 400× = 100 µm. Data are representative of three independent experiments. dpi, days post infection

Next, to monitor the dynamic pathological features and viral dissemination in the body system, minks were euthanized and systemic analyses at 0 (negative control), 1, 3, 4, 6, and 14 days dpi (*N* = 3/group) (Supplementary Fig. 1h). The affected lungs exhibited various stages of diffuse alveolar damage related to the duration of the illness. Severe pathological findings were observed at 4–6 dpi characterized by multifocal to coalescing thickened and degenerative alveolar septa, moderate to severe pneumocyte proliferation, desquamated cells and massive inflammatory cells, extensive edema or fibrin exudation in the alveolar lumina, severe perivasculitis, and vasculitis (Fig. 1a, first line). Cells positive for viral RNA and antigens were detectable to different degrees in the degenerative and desquamated bronchiolar epithelium, and between and within alveoli in the lung tissues of all minks at 1–6 dpi. A few viral RNA-positive cells were also detected in the alveoli of 2/3 minks at 14 dpi (Fig. 1b). Most periodic acid-Schiff-positive exudations were observed at 4 dpi (Fig. 1c) during the examination. Consistent with the histopathological findings, more viral RNA and viral antigens were detected in the lung lesions at 4–6 dpi (Fig. 1b, d). More robust mature collagen fibers were observed by Masson staining at 14 dpi during the later stage of illness compared to 0 dpi (Fig. 1e). Furthermore, the viral distributions and various kinds of inflammatory cells were analyzed using multiplexed immunohistochemistry (mIHC). Consistently, SARS-CoV-2 caused increased infiltration of inflammatory cells, especially activated Mac-2^+^ macrophages and myeloperoxidase^+^ (MPO) neutrophils, during the progression of the disease and induced peaked inflammation at 4–6 dpi (Fig. 1f). Interestingly, some viral S1 proteins were focally co-localized with Mac-2^+^ macrophages in the alveolar lumen (Fig. 1f). Furthermore, SARS-CoV-2 S1 protein was distributed in the proliferative (Ki67^+^, yellow color) bronchiolar epithelium and alveolar epithelium (pancytokeratin^+^, pan-CK^+^, red color), and co-localized with angiotensin-converting enzyme 2 (ACE2 + , cyan color) in the epithelium of the lung tissues (Fig. 1g). The semi-quantitative analysis of IHC and mIHC during the progression of lung lesions is summarized in Fig. 1h for intuitive visualization.

In summary, the respiratory system of minks is highly permissive to SARS-CoV-2. SARS-CoV-2-infected minks mimic signatures of lung pathology and inflammation (increased macrophage and neutrophil recruitment) seen in COVID-19 patients (Table 1).

**Table 1:** The comparative pathological features between autopsy results of patients and laboratory minks that infected by SARS-CoV-2

Organ systems	Similarities or differences	Patients	Minks
Respiratory system	Similarities	**Clinical signs:** labored breathing **Gross lesions**: swollen lung and edematous parenchyma **Histopathological changes**: diffuse alveolar damage (DAD) that are related to the course of the disease; The severe pathological findings characterized by multifocal to coalescing thickened and degenerative alveolar septum, moderate to severe pneumocytes proliferation, desquamated cells and activation of alveolar macrophages with scattered neutrophils and lymphocytes are filled in the alveolar cavities, formation of syncytial cells, intra-alveolar fibrin, and microthrombi, extensive edema or fibrin exudation filled in the alveolar lumen in infected minks at 4-6 dpi is similar to that in severe COVID-19 patients.	
	Differences	**Clinical signs** include fever and coughing.	The duration of clinical sign is relative shorter.
Extrarespiratory changes are present that differ in severity of the pathology, vary with the duration of illness, variable degree depend on the difference between individuals in COVID-19 patients and infected minks.			
Cardiovascular system	Similarities	Acute myocarditis, focal myocyte necrosis, a few interstitial monocytes, lymphocytes, and/or neutrophils infiltrates.	
	Differences	Myocardial hypertrophy, ischemic cardiomyopathy, atherosclerosis, and general interstitial fibrosis in individual patients.	Vasculitis and perivasculitis.
Hepatobiliary system	Similarities	Focal to multifocal inflammatory cells infiltration in the periportal zone, scattered or clustered hepatocyte necrosis or apoptosis, and mutifocal proliferation of bile ducts. Hepatic sinusoids congestion and infiltration of macrophages. SARS-CoV-2 viral particles were identified in the macrophage in the hepatic sinusoids.	
	Differences	Steatosis, cirrhosis, hepatic congestion in individual patients.	Individual or clustered megakaryocyte.
Urinary system	Similarities	Acute tubular injury with tubular dilation, moderate to severe interstitial nephritis, SARS-CoV-2 viral particles were identified in the tubular epithelium. Proximal tubules manifested epithelial degeneration, focal necrosis, and exfoliation.	
	Differences	Arteriosclerosis, granulomatous interstitial nephritis, focal segmental glomerulosclerosis.	Acute pyelonephritis
Endocrine system	Similarities	Adrenal gland cortex degeneration, infiltration of inflammatory cells in the adrenal gland.	
	Differences	Pancreatic islet cell degeneration. Infiltration of inflammatory cells in the interfollicular region of thyroid gland. Focal hemorrhage and necrosis in the cortex of the adrenal gland. Hyperglycemia. Diabetic ketoacidosis	Not applicable
Alimentary system	Similarities	Diarrhea. Different segmental mucosa epithelia of the alimentary system displayed different extents of degeneration, necrosis, and exfoliation.	
	Differences	Vomiting, anorexia, and abdominal pain. The gastrointestinal mucosa cells are infected by SARS-CoV-2 without obvious histopathological changes. The intestinal epithelium cells and submucosa ganglion cells are infected by SARS-CoV-2.	Focal to multifocal inflammatory cells infiltration in the mucosal and submucosal tissues. SARS-CoV-2 was localized in the granulomas of the stomach muscularis and the meninges in one infected mink.
Immune system	Similarities	The spleens and lymph nodes exhibit the lymph follicle depletion or necrosis. The red pulp of the spleen expanded and the white pulp of the spleen exhibited atrophy with significantly reduced lymphocytes.	
	Differences	Hemorrhage and anemic infarction were often found in the spleens.	Increased megakaryocyte.
Nervous system	Similarities	Meningitis, perivascular inflammatory response.	
	Differences	Subarachnoid hemorrhages, edema, cerebral spongiosis, cerebral focal necrosis.	Focal to multifocal endotheliitis, pigmented vascular.

### SARS-CoV-2 infects multiple extrapulmonary organs and systems accompanied with the inflammatory response in minks

At 0, 1, 3, 4, 6, and 14 dpi, the histopathological features of all infected minks were recorded and compared with those of patients with COVID-19 that have been reported on autopsy (Table 1). At the early stage (1 and 3 dpi), there were mild focal to multifocal inflammatory responses in the extrapulmonary organs, including the gastrointestinal tract, liver, and kidney. At 4 and 6 dpi, infected minks displayed moderate to severe inflammatory responses and different degrees of lesions in multiple organs, as observed by hematoxylin and eosin (H&E) staining. To further confirm the distribution of SARS-CoV-2 and the relationship between the virus and the lesions, all affected organs and tissues were serially sectioned and stained for viral S1 (viral antigen), SARS-CoV-2 RNA (viral RNA), Mac-2 (macrophages), MPO (neutrophils), CD3 (T lymphocytes), and CD20 (B lymphocytes). Next, CD31 (vein endothelial cells) was analyzed by immunofluorescence staining together with viral S1 and Mac-2 to observe the relationship of location.

**Fig. 2:**
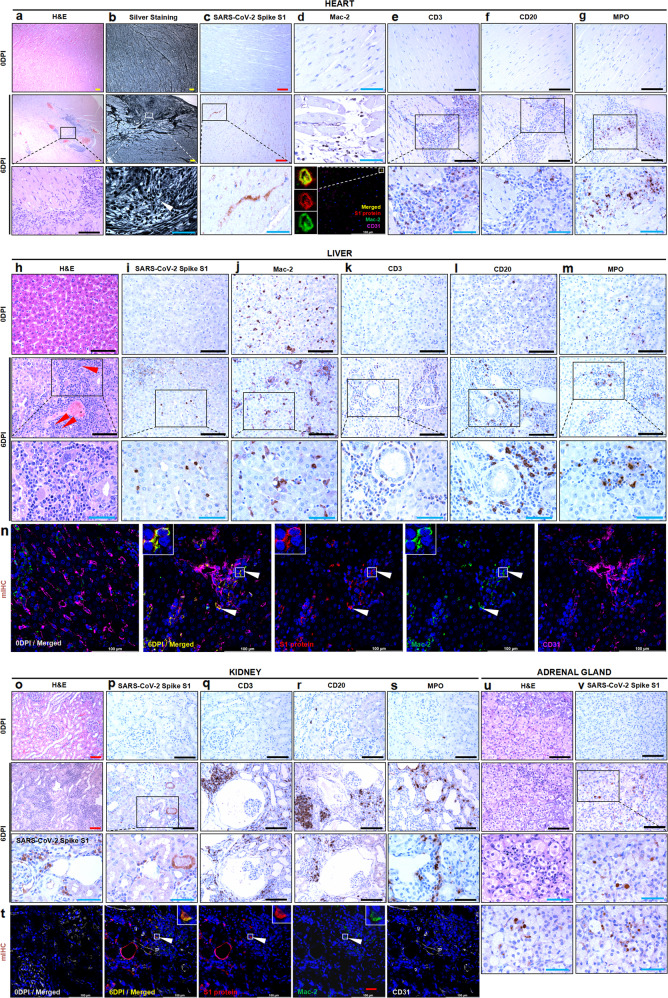
Histopathological features of the cardiac, hepatic, kidney, and adrenal gland lesions in the infected minks at 6 dpi compared with the negative-control minks (*n* = 3/group). **a** The pathological changes of the whole-heart transverse section and its magnification in an infected mink at 6 dpi compared with the normal whole-heart transverse section of a negative-control mink at 0 dpi. **b** Compared with the normal structure of nerve fibers, more fractured and entangled nerve fibers (white arrowhead) were stained by silver staining. **c** More viral S1 antigen was distributed in the myocardium of infected minks than control minks. **d** Mac-2^+^ macrophages were scattered in the interstitium of the fractured myocardium compared with that in the negative control. Viral S1 protein (red) was co-localized with Mac-2^+^ macrophages (green) in the lesion area of the heart tissues stained by immunofluorescence. Vascular endothelial cells are stained with CD31 (magenta). **e** Abundant CD3^+^ lymphocytes were stained in the serial section from the lesion area in the myocardium of infected minks compared with the negative controls. **f** A few CD20^+^ lymphocytes were stained in the serial section from the lesion area in the myocardium of infected minks compared with the negative controls. **g** Massive MPO^+^ neutrophils were stained in the serial section from the lesion area in the myocardium of infected minks compared with the negative controls. **h** Compared with normal control livers, affected livers had focal to multifocal infiltration, multinuclear syncytial hepatocytes (red arrowheads), and proliferation of bile ducts. **i** More viral S1 antigen was distributed in the hepatic sinusoids and in the areas of inflammatory infiltration around the bile ducts of the infected minks compared to controls. **j** Mac-2^+^ macrophages were scattered in the hepatic sinusoids in the serial section. **k** Massive CD3^+^ lymphocytes were stained in the serial section from the infected minks compared with the negative controls. **l** Abundant CD20^+^ lymphocytes were stained in the serial section from the infected minks compared with the negative controls. **m** More MPO^+^ neutrophils were stained in the serial section from the infected minks compared with the negative controls. **n** Viral S1 protein (red) was co-localized with Mac-2^+^ macrophages (green) (white arrowheads) in the hepatic sinusoids stained by CD31 (magenta), while no viral S1 protein was in the negative control. **o** Compared with the normal control kidneys, affected kidneys exhibited interstitial nephritis and contracted glomeruli with expansive Bowman’s capsules. **p** More viral S1 antigen was distributed in the renal tubules and infiltrate of infected minks compared to normal controls. **q** Massive CD3^+^ lymphocytes were stained in the serial section from the infected minks compared with the negative controls. **r** Abundant CD20^+^ lymphocytes were stained in the serial section from the infected minks compared with the negative controls. **s** More MPO^+^ neutrophils were stained in the serial section from the infected minks compared with the negative controls. **t** Viral S1 protein (red) was co-localized with infiltrating Mac-2^+^ macrophages (green) (white arrowheads), while no viral S1 protein was in the negative control. **u** Compared with the normal control adrenal gland, the affected tissues exhibited inflammatory cell infiltration. **v** More viral S1 antigen was distributed in the cortical cells and the chromaffin cells of infected minks than normal controls. Red scale bar at 100× = 100 µm, black scale bar at 200× = 100 µm, blue scale bar at 400× = 50 µm, white scale bar at 400×=100 µm. Data are representative of three independent experiments. dpi days post infection

Myocardial injury has been reported in more than 20 postmortem studies of COVID-19.^15,16^ Lesions in the heart attributed to SARS-CoV-2 infection included acute myocarditis, focal myocyte necrosis, focal interstitial inflammatory infiltration, vasculitis, and perivasculitis, which were more serious at 6 dpi compared to the negative-control minks (Fig. 2a). Normally, the myocardium is evenly innervated by nerve fibers via silver staining, but the serial sections of the lesion tissues in the heart at 6 dpi exhibited more severe nerve fiber breaks, malalignment, and entanglement (Fig. 2b). Viral S1 protein was scattered in a few cardiomyocytes (Fig. 2c). Mac-2^+^ macrophages were scattered in the interstitium of the disordered myocardium. Furthermore, confocal microscopy revealed that the SARS-CoV-2 S1 protein was co-localized with macrophages in the lesioned areas of the heart tissues (Fig. 2d). Vasculitis and perivasculitis were infiltrated and surrounded by abundant CD3^+^ lymphocytes, a few CD20^+^ lymphocytes, and massive MPO^+^ neutrophils; in contrast, few inflammatory cells were observed in the negative-control group (Fig. 2e–g).

Hepatic injury with metabolic disorders in the hepatobiliary system has been reported in approximately 40% of COVID-19 patients.^15,17,18^ In infected minks, the major histopathological findings were focal to multifocal inflammatory cell infiltration in the periportal zone, scattered or clustered hepatocyte necrosis or apoptosis, individual or clustered multinuclear syncytial hepatocytes, and multifocal proliferation of bile ducts (Fig. 2h). Intriguingly, viral antigen-positive cells were mainly located in the hepatic sinusoids and in the areas of inflammatory infiltration around the bile ducts (Fig. 2i). Kupffer cells were positive for Mac-2 in the hepatic sinusoids in the negative-control group and in the infected groups. Notably, Kupffer cells changed into megalocytes and clustered in infected minks (Fig. 2j). The inflammatory cells in the liver were mainly CD3^+^ lymphocytes, CD20^+^ lymphocytes, and MPO^+^ neutrophils, whereas a few inflammatory cells were observed in the livers of negative-control minks (Fig. 2k–m). SARS-CoV-2 S1 protein was consistently co-localized with Kupffer cells in the hepatic sinusoids surrounded by CD31^+^ endothelial cells (Fig. 2n).

Detailed kidney histopathology in patients with COVID-19 has been examined and reported in several autopsy studies.^19–21^ In infected minks, the major pathological changes included acute tubular injury with tubular dilation (which was also the most common finding in clinical patients with COVID-19), renal tubular epithelial cells sloughing in the lumen, moderate to severe interstitial nephritis, and acute pyelonephritis. In the cortex areas, multifocal renal corpuscles exhibited contracted glomeruli with expansive Bowman’s capsules and increased mesangial matrix volume (Fig. 2o). Consistent with previous reports in patients,^19,20^ SARS-CoV-2 RNA or S1 protein was located in the renal tubules and in the areas of inflammatory infiltration by in situ hybridization (ISH), IHC, and immunofluorescence (Fig. 2p). There were massive CD3^+^ lymphocytes and CD20^+^ lymphocytes and extensive MPO^+^ neutrophil infiltration in the renal interstitium; by contrast, only a few inflammatory cells could be detected in the kidneys of negative-control minks (Fig. 2q–s). SARS-CoV-2 S1 protein was co-localized with Mac-2^+^ macrophages in the renal interstitium (Fig. 2t).

We previously reported robust viral RNA expression in a dense distribution in hamsters infected with SARS-CoV-2.^6^ Here, we also observed multifocally clustered inflammatory cells in the adrenal cortex and medulla in infected minks at 6 dpi (Fig. 2u). SARS-CoV-2 S protein was mainly detected in the cortical cells of the zona reticularis, the chromaffin cells of the adrenal medulla, and the interstitium of the adrenal gland (Fig. 2v). Clinically, severe COVID-19 often displays critical illness-related corticosteroid insufficiency, and corticosteroids are recommended by the World Health Organization to treat patients suffering from severe cases of COVID-19.^22,23^ We once again demonstrated that the adrenal gland might be an undervalued but important organ for SARS-CoV-2 infection.

**Fig. 3:**
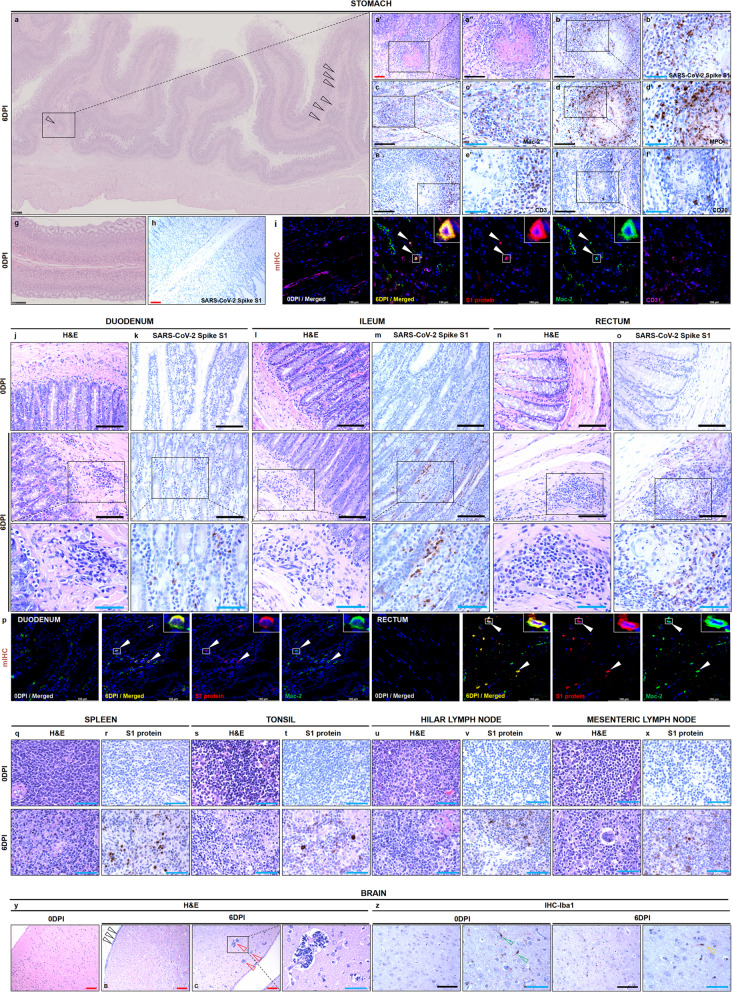
Histopathological features of the stomach, intestinal tract, lymphatic system, and brain in the infected minks at 6 dpi compared with that in the negative-control minks. **a**, **a’**, **a”** Granulomatous interstitial gastritis (black arrowheads) were observed in the infected mink at 6 dpi. **b**, **b’** Viral antigens were scattered distributed inside and outside the granulomas. **c**, **c’** Mac-2+ macrophages were stained in the periphery of the granuloma. **d**, **d’** Abundant MPO^+^ neutrophils were stained inside and outside the granuloma. **e**, **e’** Massive CD3^+^ lymphocytes were stained in the periphery of the granuloma. **f**, **f’** A few CD20^+^ lymphocytes were stained in the periphery of the granuloma. **g**, **h** The negative-control tissue was stained by hematoxylin and eosin and stained for viral S1 protein. **i** Viral S1 protein (red) was co-localized with Mac-2^+^ macrophages (green) (white arrowheads) in the granulomas. The major segments of the intestinal tract, including the duodenum (**j**), ileum (**l**), and rectum (**n**), are shown. Viral S1 protein was examined in serial sections of the duodenum (**k**), ileum (**m**), and rectum (**o**). **p** Viral S1 protein (red) was co-localized with Mac-2^+^ macrophages (green) (white arrowheads) in the lamina propria of the duodenum and a granuloma of the rectum. The major lymphatic organs include the spleen (**q**), tonsil (**s**), and hilar (**u**) and mesenteric (**w**) lymph nodes. Viral S1 protein was examined in serial sections of the spleen (**r**), tonsil (**t**), and hilar (**v**) and mesenteric (**x**) lymph nodes at 0 and 6 dpi. **y** The histopathological lesions in the brain including lymphocytic meningitis (black arrowhead) and perivascular inflammatory response (red arrowheads) were observed by hematoxylin and eosin staining. **z** Iba1 protein was visualized by immunohistochemistry. More ramified microglia (green arrowheads) were stained in the negative control, while amoeboid microglias (yellow arrowheads) were stained in the infected minks. Red scale bar at 100× = 100 µm, black scale bar at 200× = 100 µm, blue scale bar at 400× = 50 µm. Data are representative of three independent experiments. dpi days post infection

Clinical symptoms such as diarrhea, vomiting, anorexia, and abdominal pain have been recorded in the gastrointestinal tract.^24,25^ Relative to lung tissues and other organs and systems, the histopathological literature of the alimentary system is fewer in patients with COVID-19. Detailed examination and observation in minks provide a valuable reference. Notably, granulomatous interstitial gastritis and rectitis were observed in one infected mink at 6 dpi (Fig. 3a). A granuloma was formed in the muscularis mucosa of the stomach (Fig. 3a’, a’’) characterized by a necrotic center. Viral antigens were scattered inside and outside the granulomas (Fig. 2b, b’), activated macrophages and epithelioid macrophages (Fig. 3c, c’) were interspersed with massive neutrophils (Fig. 3d, d’), abundant CD3^+^ lymphocytes (Fig. 3e, e’) were localized at the periphery, and a few CD20^+^ lymphocytes were visible (Fig. 3f, f’). The negative controls are shown in Fig. 2g, h. SARS-CoV-2 S1 protein was co-localized with Mac-2^+^ macrophages in the periphery of the granuloma in the muscularis mucosa of the stomach (Fig. 3i). There have been several clinical cases of COVID-19 related to granulomatous inflammation, such as granuloma annulare^26^ and granulomatous interstitial nephritis.^27^ A recent report tried to determine the link between granulomatous diseases, like sarcoidosis, and SARS-CoV-2 infection.^28^ SARS-CoV-2 infection in minks might be a better tool for understanding the pathogenesis of granulomatous inflammation associated with COVID-19. Generally, the main finding in different segments of the gastrointestinal tract is focal to multifocal inflammatory cell infiltration in the mucosal and submucosal tissues. In some areas, vasculitis or perivasculitis is characterized by abnormal endothelium in small-sized vessels. Viral antigens were scattered throughout the lamina propria of the intestinal tracts, such as the duodenum and ileum (Fig. 3j–o). SARS-CoV-2 S1 protein was co-localized with Mac-2^+^ macrophages in the lamina propria of the duodenum and granulomas from the muscularis of the rectum (Fig. 3p).

The importance of the lymphoid/lymphatic system for resistance against pathogen infection is well-established. We collected and examined the major lymphatic organs, including the spleen, tonsil, and mandibular, hilar, and mesenteric lymph nodes. The main histopathological findings in the spleen included lymphocyte depletion, white pulp atrophy, and focal necrosis. Decreased lymphocytes, increased hemophagocytosis, focal to multifocal necrosis or apoptosis, and increased multinuclear giant cells were observed in the tonsils and lymph nodes. SARS-CoV-2 antigen was consistently detectable in all examined lymph tissues and organs to different degrees, revealing that the lymphoid/lymphatic system is also a route for SARS-CoV-2 spread throughout the body (Fig. 3q–x).

Clinical histopathological literature related to central nervous system involvement in COVID-19 is lacking and controversial.^29^ For patients, pathological changes in the nervous system are unremarkable, and the major findings were acute hypoxic injury with neuronal loss, encephalitis, meningitis, axonal degeneration, astrogliosis, and perivascular hemorrhage.^15,24^ In infected minks, nervous system findings included lymphocytic meningitis, focal to multifocal endotheliitis, and perivascular inflammatory response compared with the brain in the negative-control group (Fig. 3y). Ionized calcium-binding adapter molecule 1 (Iba1)-stained microglia demonstrated that more amoeboid microglia were present in the brains of infected minks; in contrast, more ramified microglia, which represent quiescent microglia, were in the brains of negative-control minks (Fig. 3z).

In summary, minks infected with SARS-CoV-2 mimicked the major histopathological features in multiple important organs seen in patients with severe COVID-19 (Table 1).

### Lipidomic and metabolomic profiling of sera of SARS-CoV-2-infected minks

**Fig. 4:**
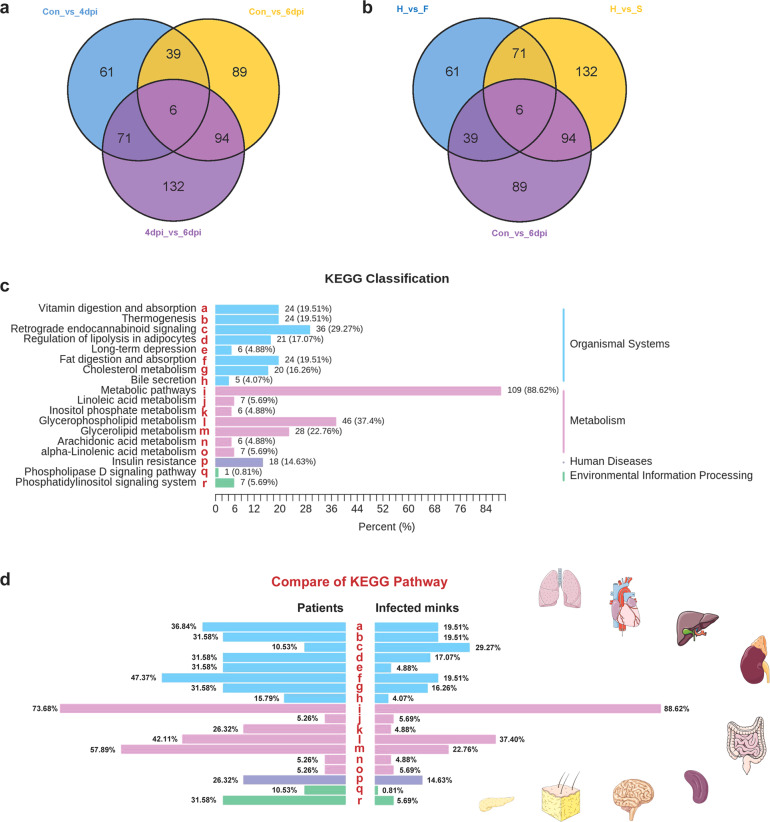
Comparison of differential metabolites between infected minks and COVID-19 patients. **a** Venn diagram among control vs. 4 dpi, control vs. 6 dpi, and 4 dpi vs. 6 dpi groups in infected minks. **b** Venn diagram among healthy vs. deceased patients, healthy vs. severe patients, and control vs. 6-dpi group. **c** Metabolome KEGG enrichment analysis of serum from infected minks. KEGG pathway analysis of differentiating metabolites in 6 dpi. **d** Comparison of KEGG pathways between infected minks and COVID-19 patients. dpi days post infection, KEGG Kyoto Encyclopedia of Genes and Genomes

**Fig. 5:**
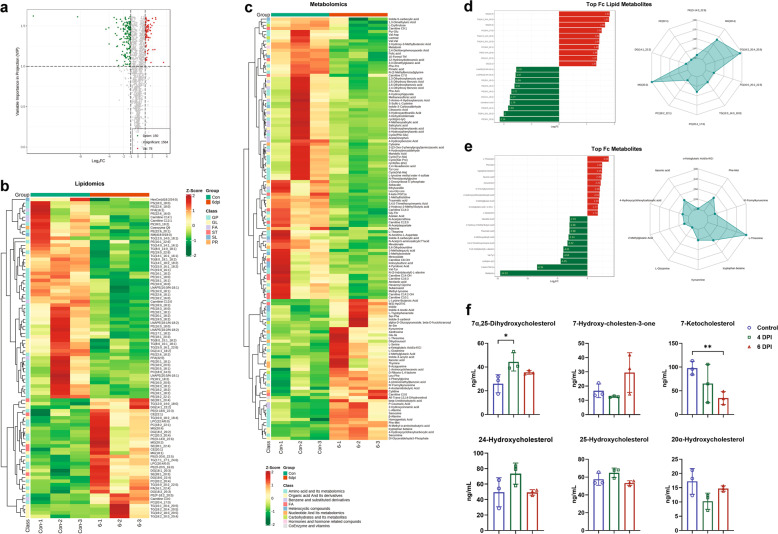
Lipidomic and metabolomic profiling of sera from infected minks at 6 dpi compared with that of control at 0 dpi. **a** Volcano plot showing the relative content differences of metabolites between minks at 0 and 6 dpi and the significance of statistical differences. Each dot in the volcano plot represents a metabolite, with green dots representing downregulated differential metabolites, red dots representing upregulated differential metabolites, and gray dots representing metabolites detected but not significantly different. **b** Cluster heatmap of differential lipidome. CE cholesterol ester, DG diacylglycerides, TG triglycerides, FA fatty acid, FFA free fatty acids, PC phosphatidylcholine, LPC lysophosphatidylcholine, PE phosphatidylethanolamine, LPE lysophosphatidyl ethanolamine, LPG lysophosphatidylglycerol, PI phosphatidylinositol, PS phosphatidylserine, LPO lipid peroxide. **c** Cluster heatmap of the differential metabolome. **d** Bar chart and radar map of differential lipidome. **e** Bar chart and radar map of differential metabolites. **f** Serum concentrations of six steroids. *P < 0.05, **P < 0.01. Data are presented as mean ± standard deviation with individual data points shown

As lipids and metabolites are important molecular constituents in body sera contributing to physiology and pathology, we performed widely targeted lipidomic and metabolomic analyses of sera from animals from the control, 4-dpi, and 6-dpi groups. Significant metabolites between groups based on log2 fold change (FC) > 1, *P* < 0.05, and variable importance in projection (VIP) ≥ 1 were screened and determined. Altogether, 1812 metabolites, including hydrophilic and hydrophobic compounds, were identified and quantified. There were 177 metabolites that changed significantly between the control and 4-dpi groups, and 228 between the control and 6-dpi groups, indicating a positive correlation between significant changes in metabolites and the course of disease in infected minks (Supplementary Table 2 and Fig. 4a). Of the 228 differential metabolites between the control and 6-dpi groups, there were 150 downregulated metabolites and 78 upregulated metabolites (Fig. 5a). The details of the metabolites are shown in Supplementary Table 1. Furthermore, lipidomic (Fig. 5b) and metabolomic (Fig. 5c) alterations were summarized by heatmap comparison. Bar charts and radar maps of differential metabolites for the top upregulated and downregulated lipids and metabolites are summarized in Fig. 5d, e, respectively. Remarkably, consistent with previous data from the sera of COVID-19 patients, we also provide evidence of significant activation of the kynurenine pathway (kynurenine, upregulated 2.22-fold; N’-formylkynurenine, upregulated 2.08-fold), which is related to macrophage activation via nicotinamide adenine dinucleotide.^30^ Importantly, dysfunction of hormones and hormone-related compounds might contribute to dysregulated metabolism. Hyperinflammation and uncontrolled oxidation play important roles in COVID-19 pathogenesis. Melatonin has significant anti-inflammatory, anti-oxidative, metabolic regulatory, and immunomodulating effects. Here, we found that melatonin was significantly downregulated 14.53-fold in the 6-dpi group compared to the control group (Fig. 5e). Melatonin has been proposed as a treatment for COVID-19, but there is still a lack of experimental and clinical data on the use of melatonin in SARS-CoV-2 infection.^31,32^ Our data support that, as an important hormone, melatonin is significantly decreased in severely infected minks. Treatment with melatonin may be a potential way to combat COVID-19. In addition, 6-keto-PGF1α, which is associated with several severe and common diseases, including atherosclerosis, diabetes, hyperlipidemia, and chronic kidney failure, was significantly decreased at 6 dpi. Dysregulated amino acids at 6 dpi included L-threonine (upregulated 3.55-fold), Phe-Met (upregulated 2.48-fold), N-(3-indolylacetyl)-L-alanine (downregulated 4.01-fold), Val-Ty (downregulated 4.04-fold), and cyclo(pro-Tyr) (downregulated 4.45-fold). Dysregulated organic acids and derivatives at 6 dpi included tryptophan betaine (upregulated 2.45-fold), itaconic acid (upregulated 2.35-fold), 4-hydroxycyclohexylcarboxylic acid (upregulated 2.02-fold), 4-methoxysalicylic acid (downregulated 2.94-fold), traumatic acid (downregulated 2.98-fold), 2-hydroxy-2-methyl butyric acid (downregulated 2.98-fold), 3,4,5-trimethoxycinnamic acid (downregulated 3.31-fold).

Furthermore, we tested the endogenous steroids in serum using electrospray ionization ultra-performance liquid chromatography (UPLC) with tandem mass spectrometry (LC-ESI-MS/MS) and multiple reaction monitoring (MRM). Of the six detectable steroid hormones, 7α,25-dihydroxycholesterol was significantly increased at 4 dpi (1.709, *P* = 0.042) (Fig. 5f). 7α,25-dihydroxycholesterol is the most effective endogenous agonist of GPR183. GPR183, a G protein-coupled receptor, binds to oxycholesterols and plays an important immunomodulatory role. 7α,25-dihydroxycholesterol promotes T-cell migration by activating GPR183 and regulates the activation, migration, and function of B cells, dendritic cells, monocytes/macrophages, T cells, and astrocytes.^33^ In this study, the changes in serum 7α,25-dihydroxycholesterol may be closely related to inflammation and immune and metabolic disorders caused by SARS-CoV-2. Next, 7-ketocholesterol was significantly decreased at 6 dpi (0.354, *P* = 0.0053) compared to the control group (Fig. 5f). 7-ketocholesterol is a cytotoxic oxysterol, which is the most common metabolite after the reaction of cholesterol and oxygen-free radicals. 7-ketocholesterol significantly increases in the organs and around blood vessels in various conditions, such as atherosclerosis and aging.^34^ Here, the decrease of 7-ketocholesterol in serum was speculated to cause in situ injury due to its direct effect on lesioned tissues by cytotoxicity.

### The altered lipidomics and metabolomics of infected minks were similar to those of severe and fatal COVID-19 patients

**Fig. 6:**
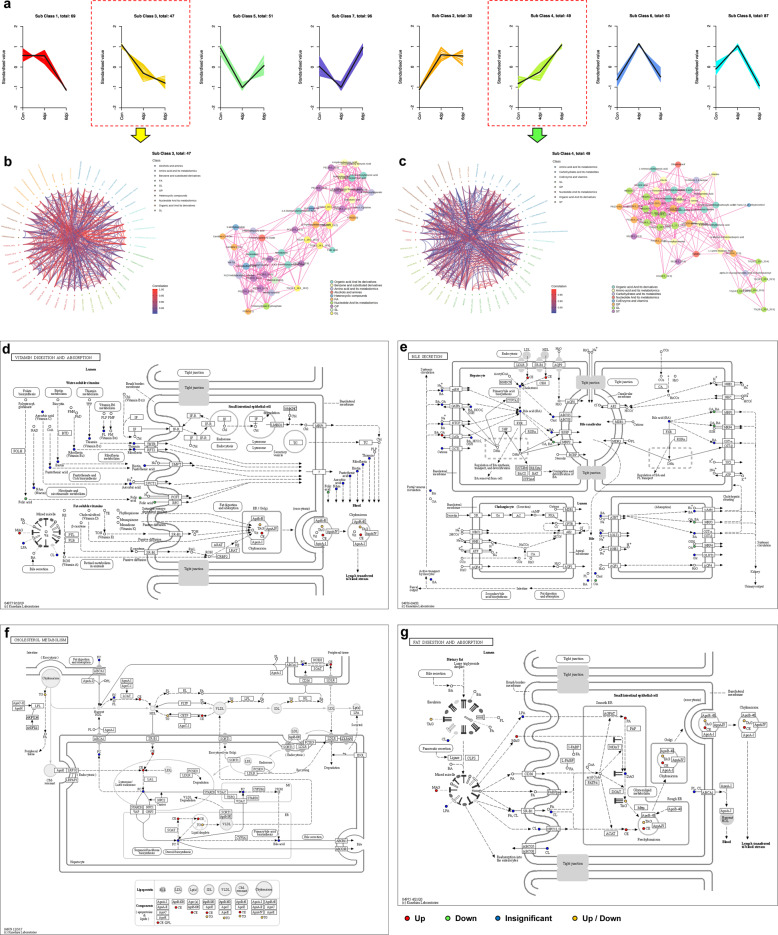
Longitudinal trajectories and differential metabolite correlation in infected minks. **a** Longitudinal trajectory clustering of significantly changed. serum metabolites. **b**, **c** Chord diagrams (left) of significant associations in metabolites in clusters 3 and 4, respectively. Two-sided t-test followed by Benjamini–Hochberg multiple comparison test. Correlation networks (right) of significant associations between metabolites in clusters 3 and 4, respectively. Nodes and edges are color-coded by molecule type, metabolic pathway, and association direction, respectively. Networks were clustered by fast greedy modularity optimization algorithm. **d**–**g** KEGG pathway map of disturbed metabolic pathways in response to SARS-CoV-2 infection in minks at 6 dpi. **d** KEGG pathway map of differential metabolites take part in vitamin digestion and absorption (KO pathway: ko04977). **e** KEGG pathway map of differential metabolites take part in bile secretion (KO pathway: ko04976). **f** KEGG pathway map of differential metabolites take part in cholesterol metabolism (KO pathway: ko04979). **g** KEGG pathway map of differential metabolites take part in fat digestion and absorption (KO pathway: ko04975). Red circles indicate significantly upregulated metabolite levels, green circles significantly downregulated metabolite levels, blue circles indicate detected metabolites that did not change significantly, and orange circles indicate metabolites that are both upregulated and downregulated. The causes of phenotypic differences among the study subjects were determined through metabolic pathways. dpi, days post infection. AAs amino acids, ApoB apolipoprotein B, BA bile acids, CE cholesterol esters, CL cholesterol, CM chylomicrons, ER endoplasmic reticulum, FA fatty acids, MAG monoacylglycerols, PL phospholipids, PA phosphatidic acid, TG triglycerides, TAG triacylglycerols

To study the variation trend of the relative contents of metabolites in different samples, the relative contents of different metabolites were standardized and k-means clustering analysis was performed. We identified eight main clusters, in which sub-class 3 (47 metabolites) showed a downward trend and sub-class 4 (49 metabolites) showed an upward trend with infection (Fig. 6a). We next characterized significant differential metabolites that were enriched in sub-class 3 (Fig. 6b) and sub-class 4 (Fig. 6c) by chord diagrams and correlation networks. Pathway analyses and network enrichment analyses of the differentially expressed metabolites are shown in Fig. 6d–g, showing enrichment in vitamin digestion and absorption (Kyoto Encyclopedia of Genes and Genomes [KEGG] pathway: ko04977), bile secretion (KEGG pathway: ko04976), cholesterol metabolism (KEGG pathway: ko04979), and fat digestion and absorption (KEGG pathway: ko04975). Folic acid (vitamin digestion and absorption); cholesterol ester (vitamin/fat digestion and absorption, bile secretion, cholesterol metabolism); 2-acylglycerol, 1-acylglycerol, and triacylglycerol (vitamin/fat digestion and absorption, cholesterol metabolism); and cholic acid, taurocholic acid, glycocholic acid (bile secretion) were significantly differential metabolites that play important roles in these networks.

Previous studies have demonstrated significant alterations in the metabolome in the plasma and sera of COVID-19 patients.^35–37^ Patients with comorbidities such as cardiovascular disease, hypertension, and diabetes, as well as elderly patients, display an even worse condition and prognosis. Although initially represented as viral pneumonia, an increasing number of cases have demonstrated that COVID-19 seems to cause a multi-system disease accompanied by lesions in several organs.^20,24,38^ Some scientists have even suggested that SARS-CoV-2-induced disease be redefined as multiple organ dysfunction with SARS-CoV-2 (MODS-CoV-2).^38^ At the beginning of the COVID-19 pandemic, more examinations in hospitals and autopsies in clinical and laboratory studies focused on the respiratory manifestations, histopathological findings in lung tissues, and how to improve respiratory symptoms. Clinicians should pay attention to SARS-CoV-2-induced pneumonia and consider potential systemic complications when treating and managing different individuals. Researchers should aim to clarify SARS-CoV-2 caused systemic pathogenicity to explore effective therapeutic schedules and individualized treatments. Therefore, minks infected with SARS-CoV-2, exhibiting both moderate to severe pneumonia and systemic damage in multiple organs similar to the findings observed in COVID-19 patients, are a reliable animal model to mimic SARS-CoV-2 infection in humans, providing a useful tool for a deeper comprehension of the underlying pathogenesis of COVID-19.

## Discussion

In our research, minks infected with SARS-CoV-2 experience infiltration by activated macrophages and neutrophils in the lung tissues and extrapulmonary organs, which is also an important pathological feature of severe COVID-19. Some studies have attempted to define the COVID-19-related systemic inflammatory response in relation to a worse prognosis.^39,40^ They proposed that COVID-19 is consistent with prior awareness of lung physiology and pathology, where local infections progress into systemic pathology related to exaggerated cytokine production (such as IL-6, TNF, and IL-1β), diffuse thrombosis, and multiorgan and system damage or failure.^39,40^ The lung-systemic loop results in further cytokine storms, increased pro-thrombotic factors, and an increased risk of death.^40^ Correspondingly, minks infected with SARS-CoV-2 provide a valuable choice as an animal model to study more effective treatment strategies focusing on severe COVID-19 patients with multiorgan injury and systemic inflammation. SARS-CoV-2 infection also induces other abnormal inflammatory and autoimmune responses, such as granulomatous inflammation, granuloma annulare,^26^ and granulomatous interstitial nephritis^27^ in patients. However, the mechanisms by which SARS-CoV-2 triggers these manifestations remain unknown. Growing evidence suggests that ACE or angiotensin II receptor I (ATR1) may play a role in increasing autoimmune reactions.^28^ In one infected mink at 6 dpi, granulomatous interstitial gastroenteritis was also observed. As the mechanism for these pathological changes has yet to be explained, further exploration of individual minks and more infected minks will provide useful data to explain clinical symptoms and help create personalized treatments for these patients. In addition, many lesions in the clinic are attributable to pre-existing injury, such as diabetic nephropathy and arteriosclerosis, making the symptoms and histopathological changes more complex.^16,24^ Therefore, minks infected with SARS-CoV-2 provide a pure and direct histopathological feature to illustrate the pathogenesis of SARS-CoV-2 infection.

Currently, the animal models reported are mainly transgenic mice, rhesus macaques, and hamsters. Pathological lesions in mice and monkeys were mainly concentrated in the lungs. The mink model of COVID-19 is similar to the hamster model and can mimic the clinical characteristics of pathological changes in multiple organs from patients. In terms of vaccine evaluation, all these animal models are suitable to be used to evaluate the vaccine protective efficiency, and due to the sensitivity and pathogenic characteristics of minks to SARS-CoV-2, they may be more suitable for studying the pathogenesis of COVID-19. Overall, multiple organ impairment induced by SARS-CoV-2 in minks needs to be further studied to determine if there is an imbalance in their function during the progression of COVID-19 and even after recovery. In addition, as an animal model of SARS-CoV-2 infection, minks could be used to explain and explore the pathogenicity, impact of mutants on viral fitness, re-infection with different mutants, immunotherapy, and vaccine efficacy on different mutants.

## Materials and methods

### Ethics statement

The animal biosafety level 3 facility at the Institute of Laboratory Animal Science was used to complete all experiments with minks (female, aged 12–24-month old). All research was performed in compliance with the Animal Welfare Act and other regulations relating to animals and experiments. The Institutional Animal Care and Use Committee of the Institute of Laboratory Animal Science, Peking Union Medical College, reviewed and authorized all protocols in this research, including research conducted in animals (SZQ20001).

### Hematoxylin and eosin staining

All collected organs were fixed in 10% buffered formalin solution, and paraffin sections (3–4 µm in thickness) were prepared according to routine practice. All tissue sections were stained with H&E. Histopathological changes in different tissues were observed using an Olympus microscope.

### Histopathology and immunohistochemistry

All collected organs were fixed in 10% buffered formalin solution, and paraffin sections (3–4 µm in thickness) were prepared as described in a previous report.^1,4,6^ Briefly, autopsies were performed according to the standard protocol of our laboratory, before sample collection, all animals were anesthetized with Zoletil^®^ 50 at 4 mg/kg (intramuscular administration), and then the main tissues were sampled and fixed in a solution of 10% neutral buffered formalin. Subsequently, paraffin sections (3–4 μm in thickness) were prepared and stained with H&E prior to observation by light microscopy. For IHC staining to identify the expression of SARS-CoV-2 S protein (GTX635654, 1:200; GeneTex, Irvine, CA) and CD138 (ZA-0584; ZSGB Bio, Beijing, China), dehydrated paraffin sections (3–4 µm in thickness) were treated with an antigen retrieval kit (AR0022; Boster Bio, Pleasanton, CA) for 1 min at 37 °C and quenched for endogenous peroxidases in 3% H_2_O_2_ in methanol for 10 min. After blocking in 1% normal goat serum for 1 h at room temperature, the sections were stained with anti-SARS-CoV-2 rabbit monoclonal antibody (GTX635654, 1:200; GeneTex), Mac-2 rat monoclonal antibody (CL8942, 1:1000; Cedarlane, Burlington, Canada), CD3 rat monoclonal antibody (ab11089, 1:500; Abcam, Cambridge, United Kingdom), CD20 rabbit polyclonal antibody (PA516701, 1:500; Invitrogen), and MPO rabbit monoclonal antibody (ab208670, 1:500; Abcam) at 4°C overnight, followed by incubation with a horseradish peroxidase (HRP)-labeled goat anti-mouse IgG secondary antibody (ZDR-5307, 1:200; ZSGB Bio), HRP-labeled goat anti-rabbit IgG secondary antibody (ZDR-5306, 1:200; ZSGB Bio), or HRP-labeled goat anti rat IgG secondary antibody (ZF-0312, 1:200; ZSGB Bio) for 1 h. Finally, the sections were visualized by incubation with 3,3′-diaminobenzidine tetrahydrochloride (DAB) and viewed using an Olympus microscope. Sequential sections from all collected tissues were directly incubated with HRP-labeled goat anti-mouse or anti-rabbit IgG and used as the omission control for different antibody staining. Sequential sections from all collected tissues were incubated with a recombinant anti-rabbit IgG antibody [SP137] (ab208334, 1:1000; Abcam) as the negative control for viral antigen expression. Mouse (*Mus musculus*), golden hamsters (*Mesocricetus auratus*), and monkey (*Macaca mulatta*) tissue sections (stored in our laboratory) were stained with different antibodies as positive or negative-control tissues (Supplementary Fig. 2).

Multiplex immunofluorescence staining was performed using a PANO IHC kit (cat. no. 0004100100; Panovue, Beijing, China).^41,42^ Different primary antibodies were sequentially applied to examine specific cell markers, including anti-SARS-CoV-2 rabbit monoclonal antibody (GTX635654, 1:200; GeneTex), Mac-2 rat monoclonal antibody (CL8942, 1:1000; Cedarlane), and CD31 rabbit polyclonal antibody (ab28364, 1:100; Abcam), followed by HRP-conjugated secondary antibody incubation and tyramide signal amplification (TSA). The slides were microwave-treated after each cycle of TSA. Nuclei were stained with 4’-6’-diamidino-2-phenylindole (DAPI; Sigma-Aldrich, St. Louis, MO) after antigen labeling. Stained slides were scanned using the Leica Application Suite X (Leica Microsystems, Wetzlar, Germany), which captures fluorescent spectra at 20-nm wavelength intervals from 420 to 720 nm with identical exposure times, and the scans were combined to build a single stacked image.

### In situ hybridization

To examine SARS-CoV-2 genomic RNA in formalin-fixed paraffin-embedded tissues, ISH was performed using the RNAscope® 2.5 HD Reagent Kit-RED (cat. no. 322310; Advanced Cell Diagnostics, Newark, CA) and the ISH Probe-V-nCoV2019-S (cat. co. 848561, positive-sense RNA probe) (genomic RNA fragment 21631-23303, RefSeq #NC_045512.2).^6^ Briefly, tissue sections were deparaffinized with xylene, underwent a series of ethanol washes and peroxidase blocking, and were then heated in antigen retrieval buffer and digested by proteinase. Sections were exposed to an ISH target probe and incubated at 40 °C in a hybridization oven for 2 h. After rinsing, the ISH signal was amplified using a pre-amplifier and amplifier conjugated to alkaline phosphatase and incubated with DAB for visualization at room temperature. Sections were then stained with hematoxylin, air-dried, mounted, and stored at 4 °C until image analysis.

### Statistical analysis

The collected data were analyzed with GraphPad Prism 8.0 software (GraphPad Software, San Diego, CA).

## Supplementary information

Supplementary Materials

Supplementary table 2

Supplementary Figure 1

Supplementary Figure 2

## Data Availability

The complete genome for this SARS-CoV-2 was submitted to NCBI (SARS-CoV-2/WH-09/human/2020/CHN, accession code MT093631.2). All raw data are available from the corresponding author upon reasonable request. Source data are provided with this paper as a source data file.
