# Supplementary material for: Integrated histopathological, lipidomic, and metabolomic profiles reveal mink is a useful animal model to mimic the pathogenicity of severe COVID-19 patients
Source: Signal Transduct Target Ther. 2022 Jan 28;7:29. doi: 10.1038/s41392-022-00891-6 (PMC8795751; doi:10.1038/s41392-022-00891-6)
Supplement: Supplementary file 1 — Supplementary Materials [file 41392_2022_891_MOESM1_ESM.docx]

Supplementary Materials for

**Integrated histopathological, lipidomic, and metabolomic profiles reveal mink is a useful animal model to mimic the pathogenicity of severe COVID-19 patients**

Zhiqi Song^1†^, Linlin Bao^1†^, Wei Deng^1†^, Jiangning Liu^1†^, Erjun Ren^3†^, Qi Lv^1^, Mingya Liu^1^, Feifei Qi^1^, Ting Chen^1^, Ran Deng^1^, Fengdi Li^1^, Yunpeng Liu^1^, Qiang Wei^1^, Hong Gao^1^, Pin Yu^1^, Yunlin Han^1^, Wenjie Zhao^1^, Junjun Zheng^4^, Xujian Liang^1^, Fuhe Yang^2*^, Chuan Qin^1*^

†These authors contributed equally to this work.

***Correspondence author**. Email: [qinchuan@pumc.edu.cn;](mailto:qinchuan@pumc.edu.cn;) yangfuhe@caas.cn.

**This PDF file includes:**

Supplementary material and methods

Supplementary Figure 1

Supplementary Figure 2

Supplementary Table 1

**Supplementary materials and methods**

**Cells and viruses**

A stock of SARS-CoV-2 virus (SARS-CoV-2/WH-09/human/2020/CHN, accession No. MT093631.2) ^1-5^ was used in this study and cultivated in Vero E6 cells maintained in Dulbecco’s modified Eagle’s medium (DMEM, Invitrogen, Carlsbad, CA) supplemented with 10% fetal bovine serum (FBS) and incubated at 37°C and 5% CO_2_.

**RNA extraction and qRT-PCR**

Total RNA was extracted from all collected organs using the RNeasy Mini Kit (Qiagen, Hilden, Germany), and reverse transcription was performed using the PrimerScript RT Reagent Kit (Takara Bio, Kusatsu, Japan) following the manufacturer’s instructions ^5-7^. qRT-PCR was performed using the PowerUp SYBR Green Master Mix Kit (Applied Biosystems, Waltham, MA), and samples were processed in duplicate with the following cycling protocol: 50°C for 2 min; 95°C for 2 min; 40 cycles of 95°C for 15 s, 60°C for 30 s, and 95°C for 15 s, 60°C for 1 min, and 95°C for 45 s. The primer sequences used for RT-PCR targeted the envelope (E) gene of SARS-CoV-2 and were as follows: forward, 5’-TCGTTTCGGAAGAGACAGGT-3’, reverse: 5’-GCGCAGTAAGGATGGCTAGT-3’. The PCR products were verified by sequencing using the dideoxy method on an ABI 3730 DNA sequencer (Applied Biosystems). During the sequencing process, amplification was performed with specific primers. The limit of RT-PCR detection is 100 copies/ml. The sequences of the primers used for this process are available upon request. The obtained sequencing reads were linked with DNAMAN (Lynnon Biosoft, San Ramon, CA), and the results were compared with the MEGALIGN module in the DNAStar software package (DNASTAR, Madison, WI).

**Preparation of homogenate supernatant**

An electric homogenizer was applied to prepare tissue homogenates by incubation in 1 mL of DMEM for 2.5 min. The homogenates were centrifuged at 825 × g at 4°C for 10 min ^3,4^.

**TCID_50_ assay**

The TCID_50_ assay was performed as follows ^3,4^. Briefly, to measure the SARS-CoV-2 titers: 10-fold serial dilutions of the virus were used to inoculate Vero cell monolayers in DMEM containing 2% FBS, which were incubated at 37°C for 4 days. Then, the cytopathic effect was observed and the TCID_50_ values were calculated using the Reed and Muench method.

**Serum preparation and extraction**

***Hydrophilic compound extraction***

Samples were sent to Metware Biotechnology Co.,Ltd (Wuhan, China) for widely targeted lipidomics and metabonomics analyses. Samples were thawed on ice, vortexed for 10 s, and mixed well. Then, 300 µL of pure methanol was added to 50 µL of serum, the mixture was vortexed for 3 min, and centrifuged at 12,000 rpm at 4°C for 10 min. The supernatant was collected and centrifuged at 12,000 rpm at 4°C for 5 min. The sample was left in a refrigerator at -20°C for 30 min, centrifuged at 12,000 rpm at 4°C for 3 min, and 150 µL of supernatant was placed in the liner of the corresponding injection bottle for on-board analysis.

***Hydrophobic compound extraction***

Samples were thawed on ice, vortexed for approximately 10 s, and then centrifuged at 3000 rpm at 4 °C for 5 min. Each sample (50 µL) was homogenized with 1 mL of the mixture (including methanol, MTBE, and internal standard mixture), and vortexed for 15 min. Then, 200 µL of water was added, the mixture was stirred for 1 min and centrifuged at 12,000 rpm at 4°C for 10 min, and 500 µL of the supernatant was kept and lyophilized. The powder was dissolved with 200 µL of mobile phase B, and then stored at −80 °C. Finally, the dissolved solution was placed in a sample bottle for LC-MS/MS analysis.

**UPLC conditions of hydrophilic compounds**

***T3 UPLC Conditions***

Sample extracts were analyzed using LC-ESI-MS/MS (ExionLC AD, Sciex, Framingham, MA; QTRAP® System, [Sciex](https://sciex.com/)). The UPLC conditions were as follows: column, ACQUITY UPLC HSS T3 C18 (1.8 µm, 2.1 mm × 100 mm; Waters, Mississauga, Canada); column temperature, 40°C; flow rate, 0.4 mL/min; injection volume, 2 μL; solvent system, water (0.1% formic acid):acetonitrile (0.1% formic acid); gradient program, 95:5 V/V at 0 min, 10:90 V/V at 11.0 min, 10:90 V/V at 12.0 min, 95:5 V/V at 12.1 min, 95:5 V/V at 14.0 min.

***Amide UPLC Conditions***

Sample extracts were analyzed using the same LC-ESI-MS/MS system as described earlier. The UPLC conditions were as follows: column, ACQUITY UPLC BEH Amide (1.7 µm, 2.1 mm × 100 mm; Waters); column temperature, 40°C; flow rate, 0.4 mL/min; injection volume, 2 μL; solvent system, water (20 mM ammonium formate and 0.4% ammonia):acetonitrile; gradient program, 10:90 V/V at 0 min, 40:60 V/V at 9.0 min, 60:40 V/V at 10.0 min, 60:40 V/V at 11.0 min, 10:90 V/V at 11.1 min, 10:90 V/V at 15.0 min.

**UPLC conditions of hydrophobic compounds**

The sample extracts were analyzed using the same LC-ESI-MS/MS system as described earlier. The UPLC conditions were as follows: column, Thermo Accucore™ C30 (2.6 μm, 2.1 mm × 100 mm; Thermo Scientific, Waltham, MA); solvent system, A: acetonitrile/water (60/40,V/V, 0.1% formic acid, 10 mmol/L ammonium formate), B: acetonitrile/isopropanol (10/90 V/V, 0.1% formic acid,10 mmol/L ammonium formate); gradient program, A/B (80:20, V/V) at 0 min, 70:30 V/V at 2.0 min, 40:60 V/V at 4 min, 15:85 V/V at 9 min, 10:90 V/V at 14 min, 5:95 V/V at 15.5 min, 5:95 V/V at 17.3 min, 80:20 V/V at 17.3 min, 80:20 V/V at 20 min; flow rate, 0.35 mL/min; temperature, 45°C; injection volume, 2 μL. The effluent was alternatively connected to an ESI triple-quadrupole (QQQ) linear ion trap (LIT) mass spectrometer (QTRAP).

**ESI-QTRAP-MS/MS of hydrophilic compounds**

T3 and amide have the same MS parameters.

LIT and QQQ scans were acquired on a QTRAP system (Sciex) equipped with an ESI Turbo Ion-Spray interface, operating in positive and negative ion modes and controlled by Analyst 1.6.3 software (Sciex). The ESI source operation parameters were as follows: source temperature, 500°C; ion spray voltage (IS), 5500 V (positive) and -4500 V (negative); ion source gas I (GSI), gas II (GSII), and curtain gas (CUR) were set at 55, 60, and 25.0 psi, respectively; and the collision gas (CAD) was high. Instrument tuning and mass calibration were performed with 10 and 100 μmol/L polypropylene glycol solutions in the QQQ and LIT modes, respectively. A specific set of MRM transitions was monitored for each period, according to the metabolites eluted within this period.

**ESI-QTRAP-MS/MS of hydrophobic compounds**

LIT and QQQ scans were acquired on a QTRAP system (Sciex) equipped with an ESI Turbo Ion-Spray interface, operating in positive and negative ion modes and controlled by Analyst 1.6.3 software (Sciex). The ESI source operation parameters were as follows: ion source, turbo spray; source temperature, 500°C; IS, 5500 V (positive) and -4500 V(negative); GS1, GS2, and CUR were set at 45, 55, and 35 psi, respectively; and the CAD was medium. Instrument tuning and mass calibration were performed with 10 and 100 μmol/L polypropylene glycol solutions in the QQQ and LIT modes, respectively. QQQ scans were acquired as MRM experiments with the CAD (nitrogen) set at 5 psi. De-clustering potential (DP) and collision energy (CE) for individual MRM transitions were performed with further optimization of DP and CE. A specific set of MRM transitions was monitored for each period, according to the metabolites eluted within this period.

**Metabolome analysis**

***Principal component analysis***

Unsupervised principal component analysis (PCA) was performed using the statistical function prcomp within R (www.r-project.org). The data were unit variance-scaled before the unsupervised PCA.

***Hierarchical cluster analysis and Pearson’s correlation coefficients***

Hierarchical cluster analysis (HCA) results of samples and metabolites were presented as heatmaps with dendrograms, while Pearson’s correlation coefficients (PCC) between samples were calculated using the cor function in R and presented as only heatmaps. Both HCA and PCC were carried out using the R package ComplexHeatmap. For HCA, normalized signal intensities of metabolites (unit variance scaling) were visualized as a color spectrum.

***Selection of differential metabolites***

Significantly regulated metabolites between groups were determined by VIP ≥ 1 and absolute log2FC ≥ 1. VIP values were extracted from the results of orthogonal projections to latent structures discriminant analysis (OPLS-DA), which also contain score plots and per-mutation plots, and were generated using the R package MetaboAnalystR. The data were log2-transformed and mean-centered before OPLS-DA. To avoid overfitting, a permutation test (200 permutations) was performed.

***KEGG annotation and enrichment analysis***

Identified metabolites were annotated using the KEGG Compound database (<http://www.kegg.jp/kegg/compound/>) and then mapped to the KEGG Pathway database ([http://www.kegg.jp/kegg/](http://www.kegg.jp/kegg/pathway.html) [pathway.html](http://www.kegg.jp/kegg/pathway.html)) (Kanehisa, M.; "Post-genome Informatics", Oxford University Press (2000)). Significantly enriched pathways were identified with a hypergeometric test p-value for a given list of metabolites.

**
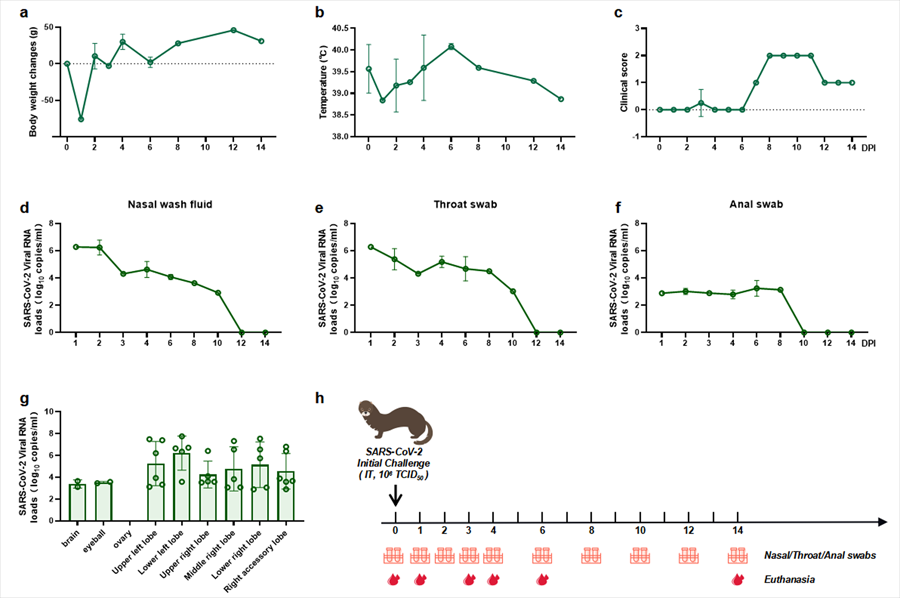
Supplementary Figure 1** Clinical features and viral load in the collected samples and organs. Clinical signs, including body weight changes (**a**), temperature (**b**), and clinical scores (**c**) (The standard for clinical scores was shown in Supplementary Table 1) in minks after viral infection during monitoring, were recorded. The viral loads of the nasal lavage fluid (**d**), throat (**e**) and anal swabs (**f**) from the infected minks were determined. **g** The viral distribution in the majority of organs and lung tissues were examined. **h** Graphical outline of sample collection and experimental design.

**
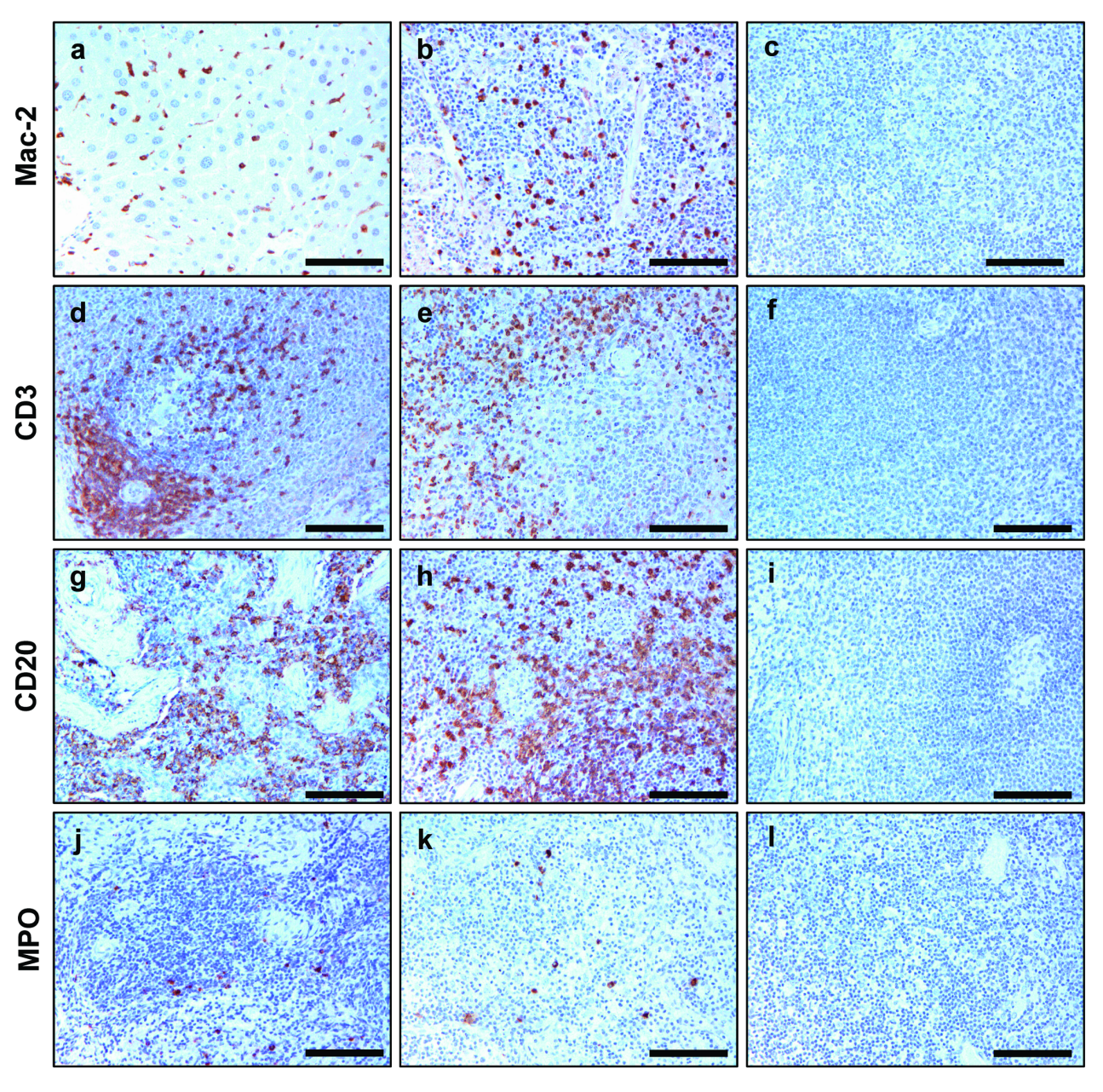
Supplementary Figure 2** Positive and negative control immunohistochemistry was performed for different antibodies used for mink tissues. **a** Mouse liver, **b** mink spleen, and **c** hamster spleen were stained to show Mac-2^+^ macrophages. **d** Cat spleen, **e** mink spleen, and **f** hamster spleen were stained for CD3^+^ lymphocytes. **g** Cat spleen, **h** mink spleen, and **i** monkey spleen were stained to show CD20^+^ lymphocytes. **j** Hamster spleen, **k** mink spleen, and **l** monkey spleen were stained to show MPO^+^ neutrophils. Black scale bar: 200×100 µm. The data are representative of three independent experiments.

**Supplementary Table 1 Ferret Clinical Sign Scoring**

| **Score** | **Nasal symptoms** | **Activity level (playfulness)** |
| --- | --- | --- |
| 0 | No symptoms | Fully playful |
| 1 | Nasal rattling or sneezing | Responds to play overtures but does not initiate play activity |
| 2 | Nasal discharge on external nares | Alert but not playful |
| 3 | Mouth breathing | Not playful, not alert |

**References**

1. Song, Z. et al. SARS-CoV-2 Causes a Systemically Multiple Organs Damages and Dissemination in Hamsters. *Front. Microbiol.* **11**, (2021).

2. Deng, W. et al. Ocular conjunctival inoculation of SARS-CoV-2 can cause mild COVID-19 in rhesus macaques. *Nat. Commun.* **11**, (2020).

3. Bao, L. et al. The pathogenicity of SARS-CoV-2 in hACE2 transgenic mice. *Nature*. **583**, 830-833 (2020).

4. Deng, W. et al. Primary exposure to SARS-CoV-2 protects against reinfection in rhesus macaques. *Science*. **369**, 818-823 (2020).

5. Gao, Q. et al. Development of an inactivated vaccine candidate for SARS-CoV-2. *Science*. **369**, 77-81 (2020).

6. Bao, L. et al. Sequential infection with H1N1 and SARS-CoV-2 aggravated COVID-19 pathogenesis in a mammalian model, and co-vaccination as an effective method of prevention of COVID-19 and influenza. *Signal transduction and targeted therapy*. **6**, 200 (2021).

7. Deng, W. et al. Therapeutic efficacy of Pudilan Xiaoyan Oral Liquid (PDL) for COVID-19 in vitro and in vivo. *Signal Transduction and Targeted Therapy*. **5**, (2020).
