# Supplementary material for: Integrated histopathological, lipidomic, and metabolomic profiles reveal mink is a useful animal model to mimic the pathogenicity of severe COVID-19 patients
Source: Signal Transduct Target Ther. 2022 Jan 28;7:29. doi: 10.1038/s41392-022-00891-6 (PMC8795751; doi:10.1038/s41392-022-00891-6)
Supplement: Supplementary file 2 — Supplementary table 2 [file 41392_2022_891_MOESM2_ESM.pdf]

Compounds	Class I	CON-1
β-Alanine	Amino acid and Its metabolomics	1662200
Ala-Ala	Amino acid and Its metabolomics	15355
Sarcosine	Amino acid and Its metabolomics	1661800
Purine	Nucleotide And Its metabolomics	12007000
Pantothenol	CoEnzyme and vitamins	1769300
2-Aminoethanesulfonic Acid	Organic acid And Its derivatives	172870000
Creatine	Organic acid And Its derivatives	3013000
Guanidinoethyl Sulfonate	Organic acid And Its derivatives	22770
Mevalonate	Organic acid And Its derivatives	440550
4-Hydroxycyclohexylcarboxylic acid	Organic acid And Its derivatives	347520
D-Mannitol	Carbohydrates and Its metabolites	104500
(R)-2-Hydroxy-3-phenylpropionic acid	Organic acid And Its derivatives	350740
Xanthosine	Nucleotide And Its metabolomics	160490
P-Aminobenzoate	Benzene and substituted derivatives	30096
3,4,5-Trimethoxybenzoic Acid	Benzene and substituted derivatives	20179
4-Hydroxyhippurate	Amino acid and Its metabolomics	12020
5-Methoxytryptophan	Amino acid and Its metabolomics	47653
2,4-Dihydroxy Benzoic Acid	Benzene and substituted derivatives	782980
Pro-Ala	Amino acid and Its metabolomics	10903
L-Methionine sulfoxide	Amino acid and Its metabolomics	165900
S-Sulfo-L-Cysteine	Amino acid and Its metabolomics	19405000
Trans-4-Hydroxy-L-Proline	Amino acid and Its metabolomics	815670
Adenosine	Nucleotide And Its metabolomics	163300
Deoxyguanosine	Nucleotide And Its metabolomics	24718
4-Acetamidobutyric Acid	Organic acid And Its derivatives	34192
Kynurenine	Amino acid and Its metabolomics	87948
tryptophan betaine	Organic acid And Its derivatives	3842300
L-Threonine	Amino acid and Its metabolomics	43935
N-(Phosphonomethyl)glycine	Amino acid and Its metabolomics	268730
Securinine	Amino acid and Its metabolomics	654350
Cis-4-Hydroxy-D-Proline	Amino acid and Its metabolomics	227870
8-Azaguanine	Nucleotide And Its metabolomics	63824
3-Ureidopropionic acid	Organic acid And Its derivatives	85564
Ile-Ser	Amino acid and Its metabolomics	333170
[illegible]C(22:6(4Z,7Z,10Z,13Z,16Z,19Z)/20:1(11Z[illegible]	GP	1968900
Hexanoyl Glycine	Amino acid and Its metabolomics	230460
2'-Deoxyuridine	Nucleotide And Its metabolomics	1766
DL-3-Phenyllactic acid(Pla)	Organic acid And Its derivatives	13607
Dl-Glyceraldehyde3-Phosphate	Organic acid And Its derivatives	11846
Jasmonic acid	Organic acid And Its derivatives	1337200
Hydroxyphenyllactic acid	Organic acid And Its derivatives	9372
Indole-3-carbinol	Heterocyclic compounds	13447
Indoleacetaldehyde	Heterocyclic compounds	855790
4-Hydroxybenzyl alcohol	Benzene and substituted derivatives	35660
(±)12-HEPE	FA	4975.5
(±)15-HEPE	FA	4975.5
(±)18-HEPE	FA	4975.5
14(S)-HDHA	FA	2087.7
Sorbitol 6-phosphate	Carbohydrates and Its metabolites	18306
Traumatic acid	Organic acid And Its derivatives	119740

2-Deoxyribose-5'-phosphate	Nucleotide And Its metabolomics	120470
L-Methionine	Amino acid and Its metabolomics	6331200
P-Coumaric Acid	Benzene and substituted derivatives	2423800
Indole-3-Acetic Acid	Heterocyclic compounds	5085300
3,4,5-Trimethoxycinnamic Acid	Organic acid And Its derivatives	10651000
Indole	Heterocyclic compounds	2230800
2-Hydroxycinnamic acid	Benzene and substituted derivatives	2423800
N1-Acetylspermine	Organic acid And Its derivatives	105390
Carnitine 2-methyl-C4	FA	995200
Mesoxalate	Organic acid And Its derivatives	1154800
Cortisol	Hormones and hormone related compunds	133780
L-Tryptophanamide	Amino acid and Its metabolomics	83800
Carnitine C20:1-OH	FA	71151
Carnitine C7:1	FA	54003
SDMA	Organic acid And Its derivatives	1828700
Phe-Met	Amino acid and Its metabolomics	10112
Leu-Phe	Amino acid and Its metabolomics	76917
Ile-Lys	Amino acid and Its metabolomics	3500.5
Ser-Phe	Amino acid and Its metabolomics	46439
Cyclo(Pro-Phe)	Amino acid and Its metabolomics	8884.2
Methyl-tyrosine	Amino acid and Its metabolomics	418390
Taurochenodeoxycholic acid	ST	957620
Taurocholic acid	ST	29778
(±)12-HETE	FA	52835
FFA(17:1)	FA	81937
FFA(18:1)	FA	7244100
FFA(20:1)	FA	271420
FFA(22:1)	FA	381950
PC(18:2_20:3)	GP	150000
PE(18:2_22:1)	GP	14226
PE(17:0_20:5)	GP	9
PE(22:6_16:0)	GP	12899000
PI(18:0_19:2)	GP	80037
FFA(32:0)	FA	30290
PE(O-20:0_22:5)	GP	4297
Carnitine C3:0	FA	12873
Carnitine C6:0	FA	6123.9
Carnitine C5-OH	FA	2847.7
Carnitine C8:0	FA	7096.4
Carnitine C4:1-2OH	FA	2107.3
Carnitine C10:1-OH	FA	4688.9
Cer(d18:0/18:0)	SL	85313
Cer(d18:0/24:1)	SL	271090
Cer(d18:1/18:1)	SL	40130
DG(18:2_22:1)	GL	24580
DG(18:1_22:4)	GL	128440
LPC(O-22:0)	GP	35447
LPC(O-14:1)	GP	10676
PC(18:2_22:1)	GP	1160200
PC(20:4_20:4)	GP	9040900
PC(O-16:1_16:1)	GP	161120

PC(O-18:0_20:4)	GP	12080000
SM(d18:0/18:0)	SL	23770000
SM(d18:2/23:1)	SL	3090600
TG(12:0_15:0_16:0)	GL	537420
TG(12:0_16:0_16:0)	GL	238610
TG(14:0_15:0_16:0)	GL	4213600
TG(15:0_16:0_16:0)	GL	1607500
TG(15:0_16:0_18:0)	GL	291770
TG(12:0_16:0_18:1)	GL	1851100
TG(15:0_16:0_16:1)	GL	17986000
TG(14:0_15:0_18:1)	GL	14030000
TG(15:0_16:0_18:1)	GL	3160800
TG(8:0_14:0_18:2)	GL	26697
TG(12:0_14:0_18:2)	GL	850920
TG(15:0_16:1_16:1)	GL	2831800
TG(14:0_15:0_18:2)	GL	28170000
TG(16:0_16:1_16:1)	GL	5124300
TG(14:0_16:0_18:2)	GL	3722200
TG(14:0_16:1_18:1)	GL	3488200
TG(16:0_16:1_17:1)	GL	166910
TG(15:0_16:0_18:2)	GL	4009000
TG(15:0_16:1_18:1)	GL	1929600
TG(16:0_17:1_18:1)	GL	1539900
TG(16:0_18:1_19:1)	GL	684150
TG(16:0_18:1_20:1)	GL	3913200
TG(16:0_16:1_22:1)	GL	99492
TG(16:0_18:1_22:1)	GL	609870
TG(18:0_22:0_18:2)	GL	87742
TG(16:0_18:1_24:1)	GL	320820
TG(16:0_18:1_26:1)	GL	64822
TG(8:0_15:1_18:2)	GL	399370
TG(10:0_14:0_18:3)	GL	73781
TG(8:0_16:0_18:3)	GL	66694
TG(8:0_18:1_18:2)	GL	1565600
TG(10:0_16:0_18:3)	GL	928250
TG(12:0_16:1_18:2)	GL	5135400
TG(15:0_16:1_18:2)	GL	21228000
TG(16:1_16:1_18:1)	GL	4017100
TG(16:0_16:1_18:2)	GL	11002000
TG(16:0_17:1_18:2)	GL	1181100
TG(16:1_18:1_22:1)	GL	109410
TG(21:0_18:1_18:2)	GL	71848
TG(16:1_17:1_24:1)	GL	12229
TG(16:1_18:1_24:1)	GL	29072
TG(18:1_18:1_22:1)	GL	369260
TG(18:1_18:1_24:1)	GL	40120
TG(18:1_20:1_22:1)	GL	40592
TG(14:1_16:1_18:2)	GL	2436200
TG(12:0_18:1_18:3)	GL	2831800
TG(15:0_16:1_18:3)	GL	1726500
TG(14:0_16:0_20:4)	GL	225740

TG(15:0_18:2_18:2)	GL	1721200
TG(16:1_17:1_18:2)	GL	282450
TG(15:0_19:2_19:2)	GL	163870
TG(17:1_18:1_18:2)	GL	1675200
TG(16:1_18:1_22:2)	GL	116380
TG(20:1_24:1_18:2)	GL	55543
TG(15:0_18:2_18:3)	GL	812630
TG(15:0_16:1_20:4)	GL	2335000
TG(16:0_16:1_20:4)	GL	1363200
TG(14:0_16:1_20:5)	GL	62565
TG(15:0_16:1_20:5)	GL	452650
TG(16:0_16:0_22:6)	GL	291220
TG(12:0_18:3_20:4)	GL	1374000
TG(16:1_16:1_20:5)	GL	294790
TG(15:0_16:1_22:6)	GL	1830500
TG(16:0_16:1_22:6)	GL	185130
TG(18:2_20:4_20:5)	GL	175020
TG(18:2_20:5_20:5)	GL	29548
DG(12:0_18:1)	GL	25102
DG(12:0_18:2)	GL	40831
DG(13:0_18:2)	GL	815710
DG(14:1_18:2)	GL	124830
DG(15:1_18:2)	GL	92533
DG(24:1_18:2)	GL	17881
Cer(d18:1/23:0(2OH))	SL	22009

CON-2	CON-3	4DPI-1	4DPI-2	4DPI-3	VIP	p_value
1001600	996970	5222100	1882800	3105300	1.523586646	0.14882971
10336	17456	47670	30558	32945	1.681101447	0.037413922
997070	948980	4993300	1756900	2865400	1.483136257	0.163403006
28553000	19866000	33281000	71551000	42655000	1.488339594	0.113216236
712870	934050	2091200	3513000	1859800	1.424790765	0.104134872
175570000	150860000	521080000	467490000	437020000	1.841655828	0.003375028
2006200	1982700	38991000	3323900	22269000	1.432128491	0.203381863
22580	23106	65113	50992	51324	1.823600425	0.019222516
393850	335690	189440	147930	124880	1.760407714	0.004982593
462670	302100	886880	1385700	964430	1.75930072	0.035286419
215170	85877	440810	112230	283260	1.069339304	0.267326934
691680	445650	2426400	552340	1385900	1.326755528	0.215969081
218580	126490	250760	982980	404600	1.432468967	0.23111255
24808	55469	120550	29918	102780	1.071907471	0.221959409
27492	20769	32426	93864	55469	1.50412679	0.167535383
58856	27823	16970	9767.8	10585	1.151627347	0.273842698
86962	91969	243880	128240	167420	1.559645217	0.075500991
2844700	1652400	1380300	238880	867630	1.001594645	0.262772382
2630.1	18237	138100	16658	105980	1.382367128	0.168617739
416170	178680	1663800	242290	684170	1.202400836	0.281930195
133350000	72263000	24558000	32614000	23063000	1.013245021	0.279834331
659220	641660	6053500	1137700	3617700	1.523043706	0.177752336
75456	87065	138770	483860	323720	1.383407508	0.166483978
86662	40026	209030	47899	116440	1.158126085	0.249876403
77085	26771	108520	87854	94373	1.50516467	0.068157386
117950	75978	462310	399910	312010	1.812164529	0.015105838
5117700	3945000	12088000	17862000	9954400	1.753675854	0.058605825
28372	9	53253	177530	68268	1.057144484	0.185426879
168160	170900	1886300	221680	1267300	1.343344199	0.197456598
766810	577530	5111600	1252100	3115800	1.602708697	0.1543488
226730	152960	938280	207770	551770	1.290273849	0.225923316
110500	46274	355040	112790	137340	1.390764776	0.233822551
83012	71668	677360	80893	403990	1.305628274	0.21649272
399860	322930	2489200	1356400	2097900	1.812472473	0.038524821
3759800	2449900	5713900	6323300	4572700	1.621502988	0.019315249
99688	179330	67693	32474	58672	1.613595756	0.082333629
1396.6	2665.2	4849.3	2743.5	4260	1.486487931	0.064399426
23753	20435	100220	20085	74359	1.318231025	0.191346719
7387	5282.6	12052	17226	29920	1.439846616	0.150317959
1182800	1522500	95199	333320	220500	1.736695242	0.001177276
6531.8	4752.2	24136	22639	21137	1.770210836	0.001265345
7149	13665	19017	32167	24950	1.531943202	0.046098998
494510	750890	861600	1912800	1502700	1.392410327	0.129515042
32094	34890	93730	61233	87962	1.761724476	0.04149172
38607	30336	5772.1	2109.2	10689	1.144028628	0.204916124
38607	30336	5772.1	2109.2	10689	1.144028628	0.204916124
38607	30336	5772.1	2109.2	10689	1.144028628	0.204916124
7288.2	9607.1	2292.3	631.14	1047.7	1.453461029	0.147528929
22115	21472	58943	25233	48923	1.491392312	0.138960381
106650	105550	676690	67533	475800	1.012319955	0.24031703

57328	112300	9	36614	45933	1.015539941	0.052556403
8176800	5291400	17768000	9034900	15067000	1.567829359	0.09242459
1704300	1458100	2917400	5916000	3520800	1.537258651	0.122215605
2644400	3998500	4411400	13568000	7474400	1.244814719	0.226693602
8501500	10011000	2078200	937420	1854000	1.783905785	0.001328997
1223400	1649500	1934000	5044600	3548200	1.310862473	0.173248014
1704300	1458100	2917400	5916000	3520800	1.537258651	0.122215605
9	57753	9	9	9	1.343969558	0.216239362
981070	732820	10061000	513870	5889100	1.052566576	0.238854772
451810	763980	381400	145660	298550	1.491863653	0.115128596
20791	59631	16170	13718	15451	1.440880223	0.231506141
40614	48236	96644	320690	175170	1.505128797	0.162062195
64616	73244	55188	24728	24019	1.506747169	0.068419565
65338	37607	509860	40540	256650	1.170356677	0.250903059
1099800	1438800	645050	820120	704880	1.686018524	0.066258654
5891.1	5366.1	24249	33890	34021	1.771350231	0.008470784
119910	90437	198770	244180	197750	1.751578156	0.004522248
16122	13500	46736	12544	17130	1.061097126	0.309941624
23727	18706	35065	108320	69281	1.30640139	0.180894575
16106	12948	682890	11730	398120	1.294685559	0.212351766
242890	322030	140110	107110	142680	1.727188563	0.05376005
698300	822930	3456600	857810	2522000	1.316491714	0.195092845
31710	27844	397850	14877	227400	1.014280242	0.239287529
104950	75110	58041	19094	39215	1.283926764	0.113710958
46785	72821	17166	44246	27113	1.507967603	0.050163034
1963000	5236300	919640	2790600	1790000	1.280322807	0.183771203
190190	266740	81476	100640	97726	1.796526813	0.024934416
861100	622190	265680	171590	307510	1.534880565	0.104390887
202660	130910	740040	172710	374000	1.29134291	0.246989425
42618	39732	18136	8176.7	12970	1.299997527	0.159443107
18633	24593	48222	56954	55526	1.03476994	0.023303393
3892200	8520700	4784700	3712300	3818600	1.226840343	0.236253453
40544	57996	27031	19998	30241	1.621082856	0.089357087
81756	55165	27084	27628	23723	1.388059335	0.183896154
3381	4621.6	11525	5327.7	8599.3	1.503444445	0.128645295
20473	19403	170480	68743	133070	1.76567896	0.068856027
7237.4	6715	20134	5425.1	15816	1.094441749	0.244932807
3963.8	3755.3	13114	3818.3	10111	1.356208318	0.181024649
4552.7	8697.5	19288	7505.6	19371	1.317798379	0.151730393
4910	3337	16537	3819.2	12280	1.335841482	0.180578533
4633.1	4688.4	59449	3546.4	39579	1.193342483	0.212938355
49403	60098	24481	26182	23643	1.75661298	0.062917965
419540	341400	136940	132560	144460	1.772709047	0.039896245
46067	49812	136740	47157	90552	1.296621719	0.21519401
73253	60653	21744	12633	24934	1.398847317	0.144594977
112250	138840	36092	65406	58101	1.714385837	0.003524994
34505	50379	90980	77754	80802	1.73131968	0.003329318
24470	15737	28933	57983	46068	1.564786154	0.064519717
1249200	1143200	2742300	3287500	3007800	1.838827357	0.005625054
10422000	9049000	26416000	11949000	19698000	1.519871123	0.140939304
203050	184590	64242	42343	100500	1.652857519	0.007243912

9493800	10945000	26357000	25488000	25712000	1.822743822	0.000960183
15480000	19632000	8503900	9875000	9269900	1.757532558	0.045652526
2221100	2627300	6575500	6544700	6787600	1.807029731	0.002052157
934250	766080	128710	183790	139020	1.795610524	0.032999114
150890	170180	102090	50183	58317	1.648633986	0.028385748
5461600	5227700	1129800	659980	961950	1.809757376	0.004446587
2522300	2068800	883030	290910	652220	1.615940512	0.014011901
499260	348430	266030	81048	191430	1.340013415	0.072203811
1183500	1344600	878250	553470	543850	1.647823511	0.037679593
20391000	20113000	3649400	3345300	3458700	1.848274476	0.00199607
16300000	15695000	3358800	2494100	2947400	1.837094194	0.001096327
4679500	4170000	2081300	708100	1421600	1.581534957	0.01248156
40683	34683	111240	208500	156210	1.779774908	0.044737566
567660	714010	1256600	1867000	1481000	1.690641882	0.028036259
3092700	2964600	537360	754670	620280	1.836494471	2.41638E-05
30500000	29665000	5846100	4456300	5038700	1.841986235	4.09862E-05
5003700	5298500	1489900	2394500	2064900	1.784104493	0.003732521
4281000	3766500	1739600	1852300	1509800	1.816814319	0.001361541
3929800	3896700	1386900	1956000	1358100	1.785058054	0.001191493
163090	166670	82414	43895	61743	1.736957166	0.010772372
5396200	5272700	1948900	807660	1384900	1.705650795	0.004092011
2854500	2545500	1212700	1202000	1210900	1.749544846	0.045198407
1780700	1691300	861640	424960	684130	1.690907627	0.005260697
1163900	983590	555580	363950	458810	1.605366854	0.05897253
5323200	4836700	2767200	1659700	2302600	1.670485407	0.010930098
346180	224220	111080	50377	79621	1.355493721	0.17672701
2177700	1555200	833840	228160	537510	1.247838043	0.172698813
312150	226980	125030	36195	98845	1.156624364	0.193958752
1198100	858100	298280	119720	222140	1.456262284	0.146700765
146330	121250	23069	12128	21734	1.733249463	0.059794401
638780	471790	134470	60675	101140	1.751648468	0.021883905
48285	56306	141580	184690	163000	1.773369268	0.004160686
42482	40512	277600	346350	322570	1.821137976	0.002033979
2202200	2005900	601970	912700	819820	1.74619424	0.013042892
980470	1023900	275810	300780	343990	1.840950287	7.95913E-05
4757900	5214400	2178600	2719600	2461100	1.824457377	0.000268619
24028000	22961000	8187600	4080700	6320300	1.764960612	0.00062593
4383200	4339600	2041700	1775700	2456800	1.785967366	0.001908521
7210100	8704200	3078000	3998400	3516000	1.776764902	0.032760742
1151200	1187300	669910	369330	504640	1.713464107	0.01574869
321600	215230	118790	49530	75712	1.391486223	0.150211779
252360	167790	105510	33456	78252	1.132784092	0.214195586
43898	29326	18829	5828.3	11400	1.167174212	0.206282744
119530	83484	35974	18352	19974	1.349371173	0.178831635
1380300	978290	641910	146950	410610	1.070901504	0.220115034
196110	157810	72859	23931	65198	1.003723228	0.236059717
87166	84924	25669	5883.8	21524	1.512540846	0.05720921
2230400	2418200	783210	1148800	985750	1.800332934	0.000921079
2641400	2801300	1170000	1206800	1145400	1.8506172	0.000602774
2087100	2009900	663930	590390	613380	1.836794866	0.005357168
83496	201100	484930	1199800	867220	1.632710351	0.075172331

3399400	2427900	1388500	713040	1066600	1.545959379	0.07983566
338270	285090	120720	157590	134110	1.792943895	0.003301958
262950	204660	92582	123730	93658	1.664331891	0.052049051
2106200	2111000	1084500	865240	893190	1.777657523	0.009055653
318910	223990	135300	59929	109310	1.276136632	0.170659682
157000	116970	41291	12147	32217	1.505463988	0.100473614
921660	748420	429720	274550	379040	1.737623842	0.002473467
2326900	2178400	521870	1246700	862760	1.66103685	0.017076815
755580	1122600	253680	597340	438110	1.578140909	0.045700907
50754	49995	117470	235430	182530	1.730322929	0.066052086
948880	719610	206150	420990	282000	1.507733355	0.089547654
69913	157780	815560	1518300	1259900	1.680788393	0.028833161
707480	1085300	151990	892550	456610	1.224966798	0.127755901
488940	415520	543490	1798400	1147900	1.458750801	0.166840479
2980300	2615500	885910	553800	742700	1.753981682	0.028169571
128490	179270	756970	1096000	945090	1.813333795	0.013614972
206540	199530	227360	718890	455020	1.389019195	0.193597131
104880	81691	131640	212980	154240	1.399686806	0.046186064
17582	24134	58040	66639	54249	1.782094626	0.001995609
26967	30865	92156	115510	85702	1.77999864	0.009049794
768010	847190	188800	164350	194800	1.847771334	0.000319625
208630	208190	106660	89969	64443	1.56671972	0.061101526
63524	101850	33591	16813	36003	1.666022411	0.021633722
57079	39269	28003	8148.9	9616.5	1.249453771	0.173128262
23450	17004	65129	15897	44469	1.016554099	0.277827573

Fold_Change	Log2FC	Type
2.789085356	1.479792087	up
2.576610193	1.365474293	up
2.665188409	1.414237524	up
2.440787078	1.287346447	up
2.184870998	1.127548101	up
2.855177248	1.51358031	up
9.223767834	3.2053562	up
2.445789996	1.290300535	up
0.395055081	-1.339874277	down
2.910221255	1.541128841	up
2.062153092	1.044151441	up
2.933087825	1.55242027	up
3.240644038	1.69628056	up
2.294474192	1.19816358	up
2.655742256	1.409115138	up
0.378147702	-1.402978245	down
2.381191964	1.251683931	up
0.470979606	-1.086263503	down
8.207024844	3.036859321	up
3.404876766	1.767602583	up
0.356571474	-1.487736803	down
5.106848409	2.352433235	up
2.904508917	1.538294259	up
2.466011915	1.30217977	up
2.106129752	1.074594319	up
4.165767926	2.05858247	up
3.092165827	1.62861769	up
4.135336578	2.048004756	up
5.553365472	2.473362344	up
4.742856571	2.24575624	up
2.794489433	1.48258472	up
2.743315896	1.45592076	up
4.837760777	2.274339431	up
5.628527596	2.492757567	up
2.030897709	1.022117576	up
0.311768123	-1.681454667	down
2.033837812	1.024204636	up
3.368180638	1.751969513	up
2.41470737	1.271848364	up
0.160548918	-2.638915155	down
3.287761425	1.717105614	up
2.222176819	1.151973617	up
2.035560801	1.025426315	up
2.36667511	1.242861671	up
0.251226689	-1.992938359	down
0.251226689	-1.992938359	down
0.251226689	-1.992938359	down
0.209194542	-2.25708288	down
2.150469358	1.104651575	up
3.675432307	1.877913951	up

0.284579694	-1.813095373	down
2.114705496	1.080456761	up
2.211557051	1.14506246	up
2.170307464	1.117899441	up
0.166976529	-2.582282771	down
2.062582048	1.04445151	up
2.211557051	1.14506246	up
0.00016549	-12.56096965	down
6.077306402	2.603432031	up
0.348271949	-1.521713816	down
0.21166469	-2.240147474	down
3.431821604	1.77897456	up
0.497270479	-1.00789731	down
5.142148992	2.362371412	up
0.496885948	-1.009013352	down
4.31274919	2.108607818	up
2.230352568	1.157271785	up
2.306891086	1.205949893	up
2.39294716	1.25878854	up
28.80315882	4.848155135	up
0.396517883	-1.33454216	down
2.757895798	1.463567949	up
7.165707697	2.841109194	up
0.499581356	-1.001208456	down
0.439236292	-1.186930833	down
0.380813382	-1.392843919	down
0.384213634	-1.380019382	down
0.399294461	-1.324475037	down
2.660938437	1.411935133	up
0.406754266	-1.297770618	down
3.716942292	1.89411629	up
0.486553755	-1.039328889	down
0.4326985	-1.208565976	down
0.469077991	-1.092100282	down
2.069335588	1.049167629	up
7.057821001	2.819222841	up
2.060892694	1.043269389	up
2.559270545	1.355732666	up
2.268909793	1.181999252	up
3.151946534	1.656243063	up
7.321304174	2.872100665	up
0.381420226	-1.390546744	down
0.401112371	-1.317921634	down
2.017873817	1.012835962	up
0.374234948	-1.417983803	down
0.420517482	-1.249762316	down
2.073746582	1.052239604	up
2.613525146	1.38599704	up
2.54393965	1.347064446	up
2.036447939	1.026054933	up
0.377368977	-1.405952268	down

2.384989606	1.253982979	up
0.469562855	-1.090609808	down
2.507595415	1.326304597	up
0.201774103	-2.30918707	down
0.376268582	-1.410165264	down
0.184643928	-2.437182272	down
0.29460846	-1.763129235	down
0.472599301	-1.081310598	down
0.451125776	-1.148398374	down
0.178721149	-2.48421773	down
0.191206953	-2.386793111	down
0.350615722	-1.512037407	down
4.663296199	2.221350069	up
2.159158582	1.110469208	up
0.215129766	-2.216720943	down
0.173669553	-2.525583242	down
0.385654555	-1.374618942	down
0.433460496	-1.206027577	down
0.415477211	-1.267158748	down
0.378625647	-1.401155957	down
0.282156167	-1.825434214	down
0.494651823	-1.015514702	down
0.39321016	-1.346627496	down
0.486763854	-1.038706054	down
0.478181779	-1.064368936	down
0.35987592	-1.474428522	down
0.368315614	-1.440985535	down
0.414869383	-1.269270903	down
0.269303582	-1.892694677	down
0.171271533	-2.545642716	down
0.196223029	-2.349433729	down
2.742975355	1.45574166	up
6.323285768	2.660674421	up
0.404331711	-1.306388739	down
0.313910428	-1.671575138	down
0.487122461	-1.037643589	down
0.272492194	-1.875713193	down
0.492484242	-1.02185053	down
0.393531057	-1.345450597	down
0.43865212	-1.188850855	down
0.377618222	-1.40499971	down
0.441501795	-1.179508792	down
0.421954759	-1.244839771	down
0.320139948	-1.643225383	down
0.439712594	-1.18536724	down
0.41109532	-1.282455146	down
0.249559436	-2.00254464	down
0.411833785	-1.279865908	down
0.425669225	-1.232195306	down
0.320717781	-1.640623752	down
5.000529063	2.322080742	up

0.419704577	-1.252553898	down
0.455305196	-1.13509417	down
0.490862735	-1.026608449	down
0.482474034	-1.051476793	down
0.461926647	-1.114264321	down
0.259944221	-1.943726014	down
0.436341739	-1.19646961	down
0.384680496	-1.378267409	down
0.397710235	-1.330210406	down
3.278530928	1.713049505	up
0.428609144	-1.222265465	down
6.925553995	2.791929481	up
0.474030403	-1.076948502	down
2.909977069	1.541007785	up
0.29387582	-1.766721437	down
5.676844732	2.505089283	up
2.411450894	1.269901432	up
2.308265354	1.206809083	up
2.6778413	1.421070463	up
2.973434824	1.572130453	up
0.225409415	-2.149380322	down
0.481993908	-1.052913184	down
0.33503162	-1.577630833	down
0.400672334	-1.3195052	down
2.009109393	1.006556119	up

Compounds	Class I	CON-1	CON-2
β-Alanine	Amino acid and Its metabolomics	1662200	1001600
Sarcosine	Amino acid and Its metabolomics	1661800	997070
Adenine	Nucleotide And Its metabolomics	118450	76098
α-Ketoglutaric Acid（α-KG）	Organic acid And Its derivatives	1392300	1120900
Lactose	Carbohydrates and Its metabolites	3596.2	6677.3
1,3-Dimethyluric Acid	Amino acid and Its metabolomics	162740	191080
Azelaic Acid	Organic acid And Its derivatives	1757600	1418700
Homogentisic Acid	Amino acid and Its metabolomics	38291	34449
Subericacid	Organic acid And Its derivatives	296720	145010
beta-Ureidoisobutyric acid	Organic acid And Its derivatives	81334	29819
Mevalonate	Organic acid And Its derivatives	440550	393850
Citraconic Acid	Organic acid And Its derivatives	293940	1036500
Itaconic acid	Organic acid And Its derivatives	33814	140030
2,4-Hexadienoic acid	Organic acid And Its derivatives	24902	105840
Methanesulfonic acid	Organic acid And Its derivatives	6193700	18596000
4-Hydroxycyclohexylcarboxylic acid	Organic acid And Its derivatives	347520	462670
N-Acetylornithine	Amino acid and Its metabolomics	313000	233780
3-Hydroxyphenylacetic acid	Organic acid And Its derivatives	1224100	5640400
Xanthosine	Nucleotide And Its metabolomics	160490	218580
2-Hydroxy-3-Methylbutanoic Acid	Organic acid And Its derivatives	11498000	12980000
N-Phenylacetylglycine	Amino acid and Its metabolomics	1092500	2472600
Atrolactic acid	Organic acid And Its derivatives	68142	20267
D-Ribono-1,4-lactone	Carbohydrates and Its metabolites	47927	75413
4-Hydroxybenzoic Acid	Benzene and substituted derivatives	1095000	4735700
4-Hydroxybenzaldehyde	Benzene and substituted derivatives	4485300	15122000
2-Methylglutaric Acid	Organic acid And Its derivatives	61575	50574
4-Hydroxyhippurate	Amino acid and Its metabolomics	12020	58856
4-Methoxysalicylic acid	Benzene and substituted derivatives	262390	1608800
Salicyluric acid	Benzene and substituted derivatives	91579	790880
Indole-3-acrylic acid	Organic acid And Its derivatives	65472	125520
4-Hydroxyphenylacetic acid	Benzene and substituted derivatives	700080	3208600
3-Dehydroshikimate	Organic acid And Its derivatives	40310	117880
2,5-Dihydroxy Benzoic Acid	Benzene and substituted derivatives	818480	2804500
5,6-Dihydrouridine	Nucleotide And Its metabolomics	138400	129030
3-Amino-4-Hydroxybenzoic Acid	Benzene and substituted derivatives	79356	320210
2,3-Dihydroxybenzoic acid	Organic acid And Its derivatives	786550	2886000
2,4-Dihydroxy Benzoic Acid	Benzene and substituted derivatives	782980	2844700
2,6-Dihydroxybenzoic acid	Organic acid And Its derivatives	761780	2753500
3-Hydroxyanthranilic Acid	Benzene and substituted derivatives	87654	238840
cyclo(pro-tyr)	Amino acid and Its metabolomics	162090	1470100
Val-Val	Amino acid and Its metabolomics	71228	91238
Val-Asp	Amino acid and Its metabolomics	37332	62112
L-Alanine	Amino acid and Its metabolomics	8021200	4962800
L-Serine	Amino acid and Its metabolomics	244160	296580
L-Glutamine	Amino acid and Its metabolomics	22676000	17927000
L-Theanine	Amino acid and Its metabolomics	2424000	1332200
N-Acetylaspartate	Amino acid and Its metabolomics	508670	321840
S-Sulfo-L-Cysteine	Amino acid and Its metabolomics	19405000	133350000
Trimethylamine N-Oxide	Alcohols and amines	41956	21046
Cytidine	Nucleotide And Its metabolomics	823490	2038200
Cytosine	Nucleotide And Its metabolomics	528370	1222300
4-Acetamidobutyric Acid	Organic acid And Its derivatives	34192	77085
Kynurenine	Amino acid and Its metabolomics	87948	117950

tryptophan betaine	Organic acid And Its derivatives	3842300	5117700
L-Threonine	Amino acid and Its metabolomics	43935	28372
3-Methyloxindole	Heterocyclic compounds	780280	417120
Securinine	Amino acid and Its metabolomics	654350	766810
4-(Aminomethyl)benzoic acid	Organic acid And Its derivatives	220840	461450
8-Azaguanine	Nucleotide And Its metabolomics	63824	110500
2-Phenylglycine	Amino acid and Its metabolomics	208350	413530
Val-Tyr	Amino acid and Its metabolomics	5384200	1098900
Glu-Ile	Amino acid and Its metabolomics	13919	16495
Pyr-Glu	Amino acid and Its metabolomics	40015	75989
Ile-Ser	Amino acid and Its metabolomics	333170	399860
Gly-Thr	Amino acid and Its metabolomics	73546	54357
cyclo(leu-phe)	Amino acid and Its metabolomics	42404	218030
Hexanoyl Glycine	Amino acid and Its metabolomics	230460	99688
4-Pyridoxic Acid	Heterocyclic compounds	101150	36402
Mandelic Acid	Organic acid And Its derivatives	289420	1260900
Sebacate	Organic acid And Its derivatives	668130	295620
9,10-DiHOME	FA	5653.4	21805
Dl-Glyceraldehyde3-Phosphate	Organic acid And Its derivatives	11846	7387
Pimelic acid	Organic acid And Its derivatives	23907	36135
Indole-3-carbinol	Heterocyclic compounds	13447	7149
L-Erythrulose	Carbohydrates and Its metabolites	52110	62047
Indoxylsulfuric acid	Heterocyclic compounds	60914	14052
N-(2-Methylbenzoyl)glycine	Amino acid and Its metabolomics	14716	27400
6-keto-PGF1α	FA	3473	1437.8
2,4-Dichlorophenoxyacetic Acid	Organic acid And Its derivatives	24550	28146
Indole-4-carboxaldehyde	Heterocyclic compounds	11226	101410
9(S)-HpOTrE	FA	12815	12689
12,13-DiHOME	FA	5653.4	21805
12-Hydroxydodecanoic acid	FA	20147	25438
Indole-3-carboxylic acid	Heterocyclic compounds	6855.3	4481.9
Acetaminophen	Benzene and substituted derivatives	3131.3	23095
L-tyrosine methyl ester 4-sulfate	Amino acid and Its metabolomics	14168	31105
3,3-Dimethylglutaric acid	Organic acid And Its derivatives	27357	35840
2-Hydroxy-2-Methyl Butyric acid	Organic acid And Its derivatives	807240	654850
Traumatic acid	Organic acid And Its derivatives	119740	106650
N-Acetyl-5-aminosalicylic acid	Benzene and substituted derivatives	13734	11905
2-Deoxyribose-5'-phosphate	Nucleotide And Its metabolomics	120470	57328
P-Coumaric Acid	Benzene and substituted derivatives	2423800	1704300
1-Methylhistidine	Amino acid and Its metabolomics	7552100	7019600
Thymine	Nucleotide And Its metabolomics	51679	86493
Melatonin	Iormones and hormone related compunc	210500	237000
All-Trans-13,14-Dihydroretinol	CoEnzyme and vitamins	21555	12416
Indole-3-Acetic Acid	Heterocyclic compounds	5085300	2644400
Indole-3-Carboxaldehyde	Heterocyclic compounds	93008	749780
3,4,5-Trimethoxycinnamic Acid	Organic acid And Its derivatives	10651000	8501500
N'-Formylkynurenine	Amino acid and Its metabolomics	41219	808200
10-Formyl-Thf	Heterocyclic compounds	14839	17767
Indole	Heterocyclic compounds	2230800	1223400
2-Hydroxycinnamic acid	Benzene and substituted derivatives	2423800	1704300
Dihydrouracil	Nucleotide And Its metabolomics	45140	30502
Phe-Pro	Amino acid and Its metabolomics	173640	239620
Mesoxalate	Organic acid And Its derivatives	1154800	451810
N-Amidino-L-Aspartate	Amino acid and Its metabolomics	54085	27058

L-Tryptophanamide	Amino acid and Its metabolomics	83800	40614
Indole-5-carboxylic acid	Heterocyclic compounds	256300	272340
N-Methyl-α-aminoisobutyric acid	Amino acid and Its metabolomics	7594400	7543000
3-Methylequuric acid	Amino acid and Its metabolomics	27497	18593
Folic acid	Organic acid And Its derivatives	14839	17767
Carnitine C16:3	FA	215860	73062
Carnitine C14-OH	FA	230300	90436
Carnitine C14:2-OH	FA	80935	39123
Carnitine C14:3	FA	72602	57814
Carnitine C8-OH	FA	113670	50745
Carnitine C7:0	FA	5802.3	8045.3
Phe-Asn	Amino acid and Its metabolomics	40236	138960
Phe-Met	Amino acid and Its metabolomics	10112	5891.1
Carnitine C9:1	FA	80047	81864
Leu-Gly-Leu	Amino acid and Its metabolomics	59178	32246
Ethylvanillin	Benzene and substituted derivatives	173810	76216
Leu-Phe	Amino acid and Its metabolomics	76917	119910
)ha-D-Glucopyranoside, beta-D-fructofurano:	Carbohydrates and Its metabolites	105600	103180
Ser-Phe	Amino acid and Its metabolomics	46439	23727
Tyr-Leu	Amino acid and Its metabolomics	62844	305400
Cyclo(Phe-Glu)	Amino acid and Its metabolomics	30520	131910
Cyclo(Ser-Pro)	Amino acid and Its metabolomics	459290	1337200
Cyclo(Tyr-Ala)	Amino acid and Its metabolomics	168590	449750
Cyclo(Val-Ala)	Amino acid and Its metabolomics	331790	1051800
N-(3-Indolylacetyl)-L-alanine	Amino acid and Its metabolomics	517000	26947
L-Lysine-Butanoic Acid	Amino acid and Its metabolomics	6043700	5933700
1-Aminocyclohexanoic acid	Organic acid And Its derivatives	40608	337120
4-Methoxysalicylic Acid	Organic acid And Its derivatives	13736	8437
Methyl-tyrosine	Amino acid and Its metabolomics	418390	242890
2-((3-Oxo-3-phenylpropyl)amino)acetic acid	Amino acid and Its metabolomics	19942	67970
FFA(16:1)	FA	177660	42358
PC(20:2/20:4)	GP	55339	58645
PC(20:3/20:4)	GP	21988	17694
PE(16:0/16:0)	GP	117730	120330
PE(16:0/18:0)	GP	74244	172070
PE(16:0/18:1)	GP	3528400	2819800
PE(16:1/18:0)	GP	17069	19219
PE(18:1/16:1)	GP	109750	99141
PE(18:2/16:0)	GP	18595000	20094000
PE(18:1/18:1)	GP	2920900	4411700
PE(18:0/18:2)	GP	12139000	16570000
PE(19:0/18:2)	GP	56745	102200
PE(20:1/18:1)	GP	46484	136590
PE(18:3/16:0)	GP	130390	196200
PE(16:1/18:2)	GP	78958	79936
PE(17:1/18:2)	GP	21687	48036
PE(18:1/18:2)	GP	8218500	17690000
PE(20:1/18:2)	GP	154550	247870
PE(18:2/22:1)	GP	14226	42618
PE(18:2/18:2)	GP	499040	1349900
PE(18:3/18:1)	GP	45876	157020
PE(18:1/20:3)	GP	49128	74268
PE(16:0/20:5)	GP	76366	185920
PE(18:0/20:5)	GP	144110	389390

PE(20:5/18:1)	GP	18610	125500
PE(22:6/16:0)	GP	12899000	3892200
PE(22:6/18:0)	GP	2652600	1216800
PE(19:0/22:6)	GP	35821	32286
PE(16:1/22:6)	GP	23663	13785
PE(22:6/18:1)	GP	841230	754360
PE(22:6/20:1)	GP	18679	13900
PE(22:6/18:2)	GP	16543	32796
PI(18:0/19:2)	GP	80037	40544
PI(18:2/18:2)	GP	18836	48771
PS(18:0/18:0)	GP	2697800	1289900
PA(16:1/22:4)	GP	92356	60129
FFA(32:0)	FA	30290	81756
PE(O-18:0/22:3)	GP	5333.8	12385
PE(O-20:5/16:0)	GP	19447	11603
PE(O-20:0/22:5)	GP	4297	3381
PE(O-14:0/22:6)	GP	4794.5	3798.9
LNAPE(16:0/N-18:1)	GP	2024400	1825700
LNAPE(18:1/N-18:2)	GP	4307300	7440900
LNAPE(18:2/N-18:2)	GP	573000	1368500
LNAPE(20:5/N-18:1)	GP	12166	68500
Carnitine C3:0	FA	12873	20473
Carnitine C13:0	FA	32608	32522
Carnitine C12:1	FA	32301	14175
Carnitine C14:1	FA	200260	72692
CE(20:1)	ST	35990	67937
CE(22:1)	ST	8898	57829
Coenzyme Q9	PR	53299	32361
DG(14:1/22:2)	GL	3479.4	3198.1
DG(18:1/20:3)	GL	31853	10687
DG(18:0/22:4)	GL	11634	7645.2
DG(18:2/20:3)	GL	30752	17810
MG(18:1)	GL	58348	83448
MG(20:2)	GL	1936.2	2492.3
HexCer(d18:2/24:0)	SL	9026.6	9198.4
LPC(20:4/0:0)	GP	256660	225890
LPC(22:4/0:0)	GP	1214100	1850000
PC(15:0/16:1)	GP	1940200	2096300
PC(18:2/22:1)	GP	1160200	1249200
PC(18:0/24:3)	GP	1439700	5932400
PC(20:4/17:0)	GP	2671300	3719500
SM(d18:0/18:0)	SL	23770000	15480000
TG(12:0/14:0/18:0)	GL	31238	19478
TG(8:0/14:0/18:1)	GL	122280	97974
TG(12:0/14:0/18:1)	GL	754050	667760
TG(8:0/16:1/18:1)	GL	495640	821040
TG(14:0/14:1/16:1)	GL	640560	570240
TG(8:0/15:1/18:2)	GL	399370	638780
TG(8:0/18:1/18:2)	GL	1565600	2202200
TG(14:1/16:1/16:1)	GL	1827700	1874200
TG(15:0/16:1/18:2)	GL	21228000	24028000
TG(14:1/18:2/18:2)	GL	4817400	5562300
TG(16:0/18:2/18:4)	GL	571730	718020
TG(15:0/16:1/22:6)	GL	1830500	2980300

TG(16:0/20:2/22:5)	GL	107630	76606
TG(17:1/17:1/24:6)	GL	182090	110110
TG(18:2/20:3/20:4)	GL	1304100	486530
TG(18:2/18:3/20:5)	GL	101650	85773
TG(16:1/20:4/20:5)	GL	127650	161000
TG(18:2/20:4/20:5)	GL	175020	206540
MG(20:4)	GL	14428	9972.3
PE(P-18:2/20:5)	GP	7525.8	10803
DG(14:1/18:2)	GL	124830	208630
DG(18:2/20:2)	GL	20093	16599
SE(28:1/20:3)	ST	103880	64905
SE(28:1/20:4)	ST	3758600	3192600
SE(28:1/22:4)	ST	21930	20866

CON-3	6DPI-1	6DPI-2	6DPI-3	p_value	Fold_Change	Log2FC	Type
996970	3265200	3216200	2068800	0.033035885	2.335628843	1.223811032	up
948980	3068600	3009300	2056900	0.023908365	2.254750059	1.172967518	up
138870	63877	26102	71932	0.074257193	0.485609655	-1.042130989	down
687690	3626900	4770500	2472100	0.050786392	3.3957743	1.763740574	up
4108.5	9	4123	9	0.118911407	0.287929356	-1.796213207	down
204450	68404	161640	39693	0.10879974	0.483165852	-1.049409601	down
1338500	331480	598310	352940	0.003645003	0.284116683	-1.815444548	down
11681	59717	62108	47212	0.056100914	2.002309852	1.001665244	up
201360	30319	90573	63081	0.05929232	0.286076599	-1.805526605	down
36758	107220	155470	109680	0.029304645	2.517527432	1.332007499	up
335690	97671	168220	128290	0.003417273	0.336880924	-1.569689359	down
521430	63421	238750	145730	0.161172989	0.24186417	-2.047731034	down
45337	101380	682160	332140	0.215434038	5.090222236	2.347728645	up
71600	10263	28912	28943	0.19075151	0.336647854	-1.570687828	down
10517000	3995700	7625400	4732400	0.218583046	0.463184042	-1.110342547	down
302100	1680800	1479900	1335200	0.002470463	4.042021415	2.015076965	up
240650	67200	88460	74732	0.014014993	0.292587278	-1.773061056	down
3070500	261910	988560	494950	0.163448447	0.175683946	-2.508945734	down
126490	327120	496410	291750	0.067762852	2.206028958	1.141451729	up
9928600	1710100	3500800	2064800	0.002060178	0.211462336	-2.241527372	down
1614600	349670	523210	371820	0.080018558	0.240303492	-2.057070479	down
30813	8265.3	26939	9766.7	0.224496759	0.377203872	-1.40658361	down
61450	132010	141730	114640	0.003761801	2.101737107	1.071582223	up
4612100	522750	1021700	1571000	0.171071055	0.298334738	-1.744996121	down
10783000	1701000	3327100	2700400	0.131164741	0.254308118	-1.975350578	down
57043	202570	317210	127790	0.10099338	3.827426829	1.936374797	up
27823	6766.3	19617	9425.9	0.26369425	0.362812187	-1.462705179	down
702900	60445	125310	106540	0.194714374	0.113552751	-3.138565441	down
357220	28246	93735	51359	0.222448075	0.13982652	-2.838290086	down
105160	266260	322710	185770	0.040236201	2.616021502	1.387374399	up
1706100	171270	575760	269690	0.167005205	0.181079223	-2.465307073	down
66486	14296	24169	16849	0.129226805	0.24619452	-2.022129445	down
1563500	103240	1010800	487610	0.163795055	0.308812528	-1.695196814	down
107870	53338	72001	59279	0.006951407	0.49192113	-1.02350107	down
132430	60659	79217	45335	0.253172557	0.348143595	-1.522245613	down
1520700	110870	968830	466390	0.176815685	0.297711452	-1.748013375	down
1652400	121220	958410	447300	0.160054408	0.289186906	-1.789925863	down
1623900	100160	941250	458770	0.158489183	0.291910383	-1.77640257	down
149850	56699	65037	55038	0.149943752	0.371105756	-1.430097719	down
731080	25146	36678	46589	0.185530392	0.045874149	-4.446174796	down
70289	40663	39218	33677	0.020586458	0.487886404	-1.035382814	down
35839	11379	35265	12965	0.093829736	0.440624469	-1.182378482	down
4681000	15758000	15235000	11079000	0.013838874	2.381658647	1.251966653	up
166640	503770	626710	350500	0.065544104	2.093613051	1.065994823	up
13825000	58714000	79742000	42941000	0.051152464	3.332788271	1.736729667	up
1355400	859010	787980	540500	0.105092502	0.42794624	-1.224498523	down
388210	46196	134530	117540	0.016106663	0.244737101	-2.03069527	down
72263000	26238000	20255000	20682000	0.250840417	0.298531673	-1.744044091	down
31595	6271.4	8390.6	9	0.033100206	0.155089485	-2.688827221	down
2202100	4093500	3746000	5642900	0.021028516	2.662511676	1.412787852	up
1041700	178870	449710	311920	0.082767506	0.336810666	-1.569990268	down
26771	108460	89194	93150	0.068800291	2.106542652	1.074877127	up
75978	332650	648230	332900	0.080114545	4.660843775	2.220591157	up

3945000	27112000	24860000	18376000	0.016343991	5.451220457	2.446579267	up
9	167230	510420	171100	0.14905388	11.73668344	3.552952884	up
578050	296890	315380	248910	0.096361852	0.485048861	-1.043798012	down
577530	1635300	1536800	1376800	0.001205509	2.275940741	1.186462995	up
313970	711900	719610	694520	0.031689721	2.134011202	1.093567749	up
46274	77639	273500	162680	0.220817479	2.329209694	1.219840528	up
268760	800660	720000	666440	0.0064751	2.45564987	1.296104874	up
2318800	66190	287540	181000	0.162835947	0.060751656	-4.040932457	down
14946	28475	52728	19342	0.205497343	2.216600529	1.148348794	up
48122	10907	37396	22574	0.087434407	0.431845046	-1.211414356	down
322930	1187300	528680	727590	0.139093413	2.314074397	1.210435247	up
62543	24866	38237	15503	0.01322389	0.41274692	-1.276670644	down
145680	11225	54237	38688	0.181239004	0.256455084	-1.963221923	down
179330	20845	95474	65214	0.083353685	0.356311754	-1.488788018	down
52708	23133	15232	18802	0.148238715	0.300467781	-1.734717795	down
827290	37099	166000	108510	0.131490172	0.131059762	-2.931703281	down
522050	31885	114370	72268	0.05404022	0.147074303	-2.765382892	down
17759	6715.8	6032.2	7382.9	0.226371981	0.445202511	-1.167466367	down
5282.6	24116	22616	21136	0.008348022	2.768359738	1.469031428	up
34857	9334.7	13402	11677	0.026824273	0.362635012	-1.463409873	down
13665	49410	14520	23281	0.231114426	2.54548904	1.347942854	up
64465	7316	37772	16244	0.034671968	0.34336196	-1.542197879	down
36400	6659.1	13463	10471	0.182613438	0.27470772	-1.864030638	down
27563	3277.8	7639.8	6314.6	0.044623692	0.247308371	-2.01561702	down
4119.3	9	9	9	0.065446735	0.00299	-8.385638747	down
28789	6853.9	3544.6	4648.4	0.000286666	0.184658526	-2.437068218	down
59779	14283	12795	15363	0.238229517	0.246156077	-2.022354739	down
14291	31598	20913	28601	0.046264424	2.038246011	1.027328191	up
17759	6715.8	6032.2	7382.9	0.226371981	0.445202511	-1.167466367	down
26326	7188.8	8674	2987.8	0.002480171	0.26213792	-1.931602028	down
2352.2	2248	2660.4	1179.4	0.182313633	0.444709045	-1.169066347	down
11510	9	4132.9	854.82	0.195700424	0.132411498	-2.916899694	down
23505	5804	8066.9	6961.6	0.079974974	0.302894821	-1.723111184	down
34745	8562.1	12251	9694.9	0.006684985	0.311490474	-1.682740052	down
733220	44749	153020	80572	0.000497221	0.126788927	-2.979499337	down
105550	8247.7	15401	18496	0.0001571	0.126964813	-2.977499372	down
8604.4	5131.2	6765.1	4899.4	0.046089967	0.490479917	-1.027734028	down
112300	33277	45680	32959	0.089838341	0.385786872	-1.374124044	down
1458100	6177800	5123300	3910000	0.024839465	2.722978053	1.445185354	up
8157800	2004500	2550200	2172300	0.000823686	0.295958996	-1.756530785	down
40088	76843	275530	146510	0.202961816	2.798625603	1.484718498	up
191090	9	9	9	0.003884819	4.22806E-05	-14.52964293	down
20492	37717	47222	66008	0.048390662	2.771551328	1.470693726	up
3998500	10763000	7066600	8862200	0.022962804	2.275865009	1.186414988	up
405120	88272	95102	81776	0.226248057	0.212475599	-2.234630924	down
10011000	707810	1261000	962410	0.003470444	0.100509884	-3.314590715	down
434790	1762500	1869000	1790200	0.022929085	4.221820592	2.077865272	up
17116	9619.5	7956.3	5648.3	0.004534192	0.467078959	-1.098261639	down
1649500	4179600	2787400	3268900	0.03158837	2.005584184	1.004022524	up
1458100	6177800	5123300	3910000	0.024839465	2.722978053	1.445185354	up
20535	61346	103600	35285	0.217006394	2.081901078	1.05790152	up
227130	82590	120270	99743	0.01542418	0.47252924	-1.081524489	down
763980	216350	365030	318330	0.131774626	0.37952999	-1.397714203	down
16460	13124	8800.3	11072	0.19311633	0.338066453	-1.564621234	down

48236	243720	125000	172820	0.055739689	3.13663481	1.64921757	up
274060	35627	229690	116190	0.128709271	0.475279681	-1.073151369	down
8901500	17475000	16702000	17266000	0.000355597	2.139989767	1.097603898	up
17806	8049.4	5702.7	10945	0.03440314	0.386520283	-1.371383972	down
17116	9619.5	7956.3	5648.3	0.004534192	0.467078959	-1.098261639	down
116930	32515	95574	66434	0.232198514	0.479295408	-1.061012976	down
148400	46142	112380	73301	0.180929465	0.494148818	-1.016982504	down
52643	19580	34813	19454	0.10058049	0.427600303	-1.225665218	down
61430	28582	41891	24903	0.009667258	0.497148755	-1.008250501	down
73222	26069	37051	36627	0.125050929	0.419745242	-1.252414124	down
5887.3	1956.7	2331.7	4861	0.041727003	0.46361522	-1.109000166	down
82667	27759	59192	33423	0.235643673	0.459683117	-1.121288415	down
5366.1	37131	41878	39820	9.51094E-05	5.560760347	2.475282162	up
80216	34857	53398	31657	0.025856704	0.495244231	-1.013787924	down
48604	11345	22131	18668	0.049236093	0.372382666	-1.425142175	down
125010	12934	40117	18374	0.059531369	0.190448384	-2.392528051	down
90437	256900	277440	236840	0.000754865	2.684568898	1.424690432	up
86380	513680	197030	267270	0.14065842	3.313389348	1.728307741	up
18706	117800	52665	70998	0.103763404	2.716974975	1.442001278	up
207650	17800	105600	34787	0.177190489	0.274680757	-1.864172249	down
78974	12436	25613	11138	0.157112589	0.203753873	-2.295100611	down
995340	118370	430820	226680	0.106307359	0.277907322	-1.847324247	down
303150	47547	139960	78803	0.102937209	0.288999338	-1.790861907	down
712560	93263	404900	220630	0.145026132	0.342911051	-1.544093695	down
184320	19572	12298	13405	0.255693336	0.062168133	-4.007680927	down
7189100	18121000	13311000	13472000	0.026190663	2.342837764	1.228257054	up
135460	324040	491100	384550	0.102569657	2.33772029	1.22510232	up
13395	9	4615.6	9	0.011259874	0.130274404	-2.940374441	down
322030	119810	205150	142630	0.057683596	0.475526538	-1.072402238	down
43602	2949.7	15105	12154	0.127259577	0.2296995	-2.122180381	down
97980	42202	43455	42534	0.248398344	0.403118888	-1.310722715	down
36986	71066	143420	122380	0.091009051	2.231343976	1.157912931	up
24959	32011	70347	49326	0.115453317	2.346560233	1.230547493	up
108390	40517	75490	43224	0.022386183	0.459607447	-1.121525921	down
119000	52047	73820	40452	0.132243885	0.45527683	-1.135184054	down
3274800	1038300	2108900	1438400	0.015081456	0.476524992	-1.069376214	down
14340	5427.2	5448.8	9546.9	0.006909235	0.403391404	-1.309747754	down
86757	38440	64617	42017	0.00988531	0.490698398	-1.027091533	down
20313000	5380200	10362000	6146900	0.009067168	0.370989119	-1.430551221	down
3735700	1094800	2073100	1455900	0.018967142	0.417751597	-1.259282752	down
16094000	4527400	7154300	4670600	0.007925106	0.364982256	-1.454101769	down
70206	31782	35848	27270	0.075475736	0.414137403	-1.271818589	down
80752	31392	40176	32041	0.177627809	0.39271717	-1.348437421	down
152500	49674	87390	70347	0.024145359	0.432927007	-1.207804291	down
60748	26908	33808	41216	0.009281258	0.464082461	-1.107546918	down
32590	8120.8	26545	11188	0.125393636	0.448171787	-1.157876264	down
13910000	3406500	5929800	4646300	0.08016129	0.351158381	-1.509806226	down
200670	48369	86643	65776	0.025007924	0.332932067	-1.586700264	down
39732	13081	13638	11624	0.163785119	0.397024105	-1.332701491	down
991870	227470	377790	255150	0.111205421	0.302874884	-1.723206146	down
123840	23816	45458	42950	0.157294871	0.343469957	-1.54174418	down
47430	15519	35199	20826	0.040958464	0.418812125	-1.255624886	down
109070	56095	31751	59769	0.140567471	0.397502666	-1.330963559	down
256260	115310	118490	130290	0.182817987	0.461013472	-1.117119183	down

80421	17232	25342	20383	0.223738667	0.280393353	-1.834475944	down
8520700	3782000	4071800	3962600	0.22568108	0.466831806	-1.099025236	down
1689200	886680	945050	834480	0.149228951	0.479654949	-1.059931152	down
35305	22654	11950	14834	0.02090903	0.478068309	-1.064711321	down
15143	11858	9	9	0.05710639	0.225818106	-2.146766931	down
787250	259090	410540	302480	0.002300074	0.407962767	-1.293490605	down
14849	4979.2	8495	7505.6	0.010265753	0.44235051	-1.176738108	down
30042	9201.9	9174.4	7642.5	0.069819514	0.327771129	-1.609239311	down
57996	21053	29289	11179	0.058099017	0.344506851	-1.53739542	down
25540	12257	8969.6	16785	0.173944849	0.408081849	-1.293069552	down
1980000	1025000	990690	934420	0.131178364	0.494346231	-1.016406263	down
86979	144340	196610	201590	0.01565809	2.26564327	1.179920724	up
55165	32053	22230	20538	0.168087802	0.447464581	-1.160154605	down
8450.4	16337	20684	17512	0.023735697	2.083861945	1.059259703	up
15931	30109	50831	32828	0.062747905	2.421574679	1.275945495	up
4621.6	8675.3	9050	7526.8	0.002178002	2.053083027	1.037791972	up
4829	7606	17000	10381	0.121092882	2.606612826	1.382176308	up
1932300	543200	1261500	826580	0.030069104	0.455049806	-1.135903634	down
5208600	1546300	2671500	2038200	0.049353967	0.368937535	-1.43855152	down
788470	238570	410110	271270	0.120206771	0.336981725	-1.56925774	down
28638	13721	7767.2	15913	0.287146826	0.34217595	-1.547189734	down
19403	41230	30624	40508	0.011697527	2.13012569	1.09093856	up
40014	4996.4	13678	11850	0.002398351	0.290310431	-1.784331683	down
28271	8087.4	16220	10047	0.11870992	0.459609081	-1.121520791	down
147370	27812	104110	58556	0.166110361	0.453171616	-1.141870593	down
65673	83695	144390	121940	0.055848548	2.063826651	1.045321798	up
32178	65343	110060	76234	0.059653737	2.544229311	1.347228706	up
43364	23867	17776	18655	0.052341212	0.46733941	-1.097457393	down
5468.1	7648.5	9385.4	14361	0.072402255	2.584878475	1.370096455	up
26100	42702	63920	42793	0.047419158	2.176791958	1.122203532	up
6513.7	13831	23648	18651	0.049804065	2.176180267	1.121798069	up
35439	44859	72678	63190	0.037195977	2.151486292	1.105333648	up
71423	77628	199840	149390	0.178070589	2.001969806	1.001420215	up
1719.4	4109.3	12325	5491.2	0.173221564	3.566339726	1.834444139	up
13747	5352.5	2036.5	7804.4	0.070310131	0.475209558	-1.07336424	down
240110	472810	598290	498510	0.014172856	2.171989594	1.119017191	up
1597600	2587500	4098900	3108600	0.045296892	2.101164811	1.071189329	up
2000600	977690	954180	871760	9.0474E-05	0.464400126	-1.106559732	down
1143200	2558000	4045300	2951900	0.045365149	2.68963576	1.427410811	up
3626300	1739700	1482300	1552900	0.250589206	0.434144967	-1.203751237	down
3229400	10957000	7617400	9327400	0.016972562	2.900334712	1.536219404	up
19632000	7955200	9852100	8721400	0.03999429	0.450540063	-1.150272695	down
9560.4	44322	54889	66527	0.01724872	2.749633356	1.459239258	up
112060	47701	27574	39995	0.001593069	0.346870731	-1.527529985	down
252970	210250	300460	275420	0.19251936	0.469392995	-1.091131783	down
695930	345760	151370	212510	0.024882513	0.352596877	-1.5039084	down
493500	293850	200330	251020	0.00540745	0.437246964	-1.193479729	down
471790	292290	130560	240830	0.036390824	0.439540644	-1.185931518	down
2005900	716900	321520	406730	0.005090715	0.250298769	-1.998276902	down
1802100	997190	577350	783880	0.011257153	0.428492006	-1.222659806	down
22961000	12703000	8533900	9654700	0.002088025	0.452843133	-1.142916715	down
5546000	2506200	1966300	2118900	0.000945618	0.413884476	-1.272699958	down
748950	1129400	1655700	1312200	0.035254565	2.009761122	1.007024035	up
2615500	1688500	711480	1080100	0.042714666	0.468615596	-1.093523126	down

87077	166250	305890	271700	0.058509889	2.741630515	1.455034155	up
173210	327860	468500	376270	0.014088138	2.519563396	1.333173757	up
894120	2891100	1009200	1910400	0.185903843	2.164335599	1.113924219	up
94987	306340	93397	185290	0.243046704	2.071551999	1.050712034	up
149610	619070	177820	307940	0.231221463	2.52094647	1.333965484	up
199530	1238100	176870	618140	0.256389671	3.498786763	1.806854741	up
12523	18959	59655	37964	0.150899186	3.157301758	1.658692152	up
9988.6	23349	14452	20504	0.04861382	2.058981404	1.0419308	up
208190	72454	41559	44407	0.032937546	0.292476692	-1.773606439	down
23211	27485	57598	40023	0.124667595	2.08847637	1.06245082	up
109710	177650	236430	168300	0.021901453	2.091168603	1.064309385	up
2926500	6557400	6220400	8471600	0.022712885	2.151249785	1.105175048	up
29441	36754	74071	44215	0.129889674	2.146268533	1.101830592	up
