# Supplementary figures and images for: Integrated histopathological, lipidomic, and metabolomic profiles reveal mink is a useful animal model to mimic the pathogenicity of severe COVID-19 patients
Source: Signal Transduct Target Ther. 2022 Jan 28;7:29. doi: 10.1038/s41392-022-00891-6 (PMC8795751; doi:10.1038/s41392-022-00891-6)

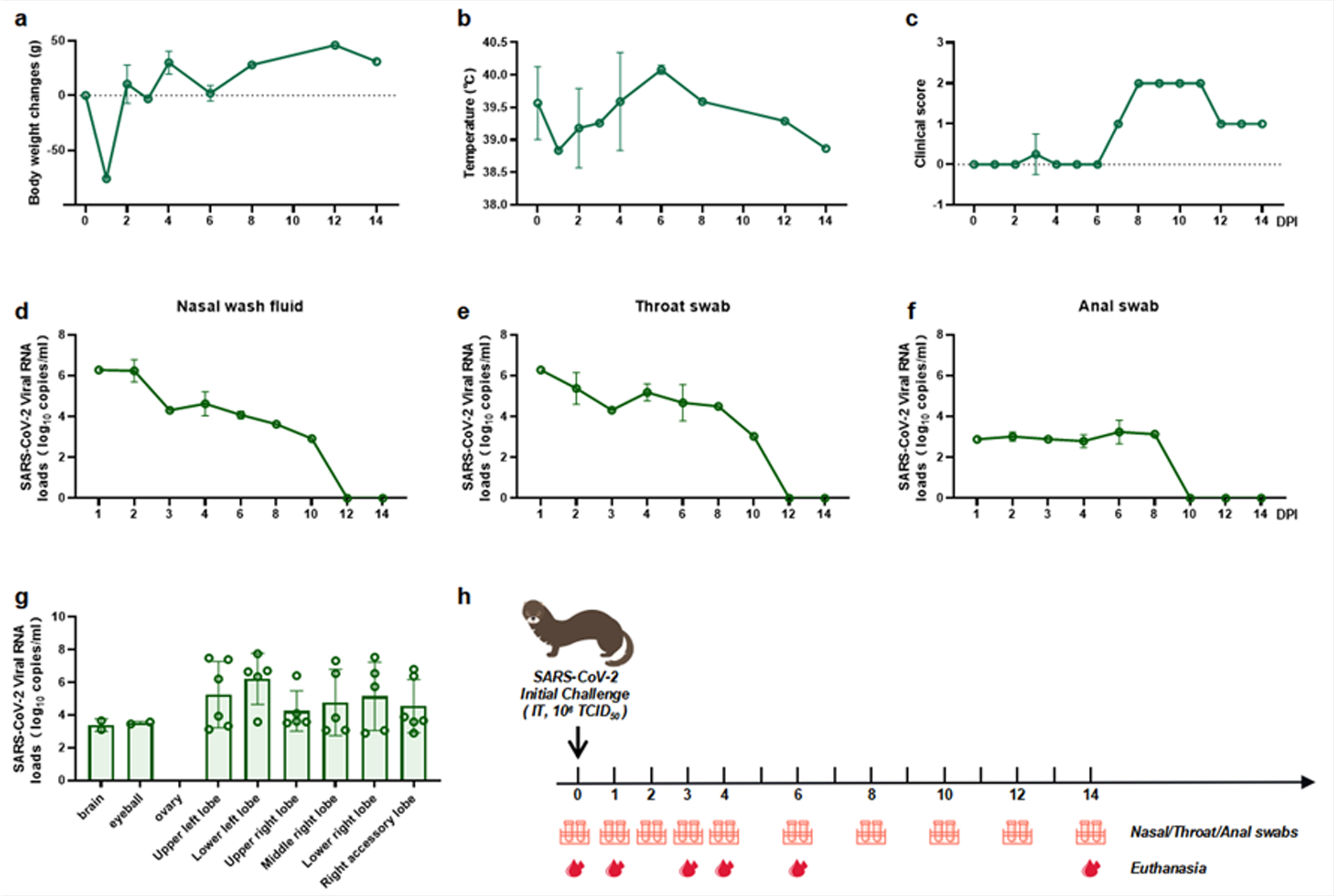

Supplement: Supplementary file 3 — Supplementary Figure 1 [file 41392_2022_891_MOESM3_ESM.tif]

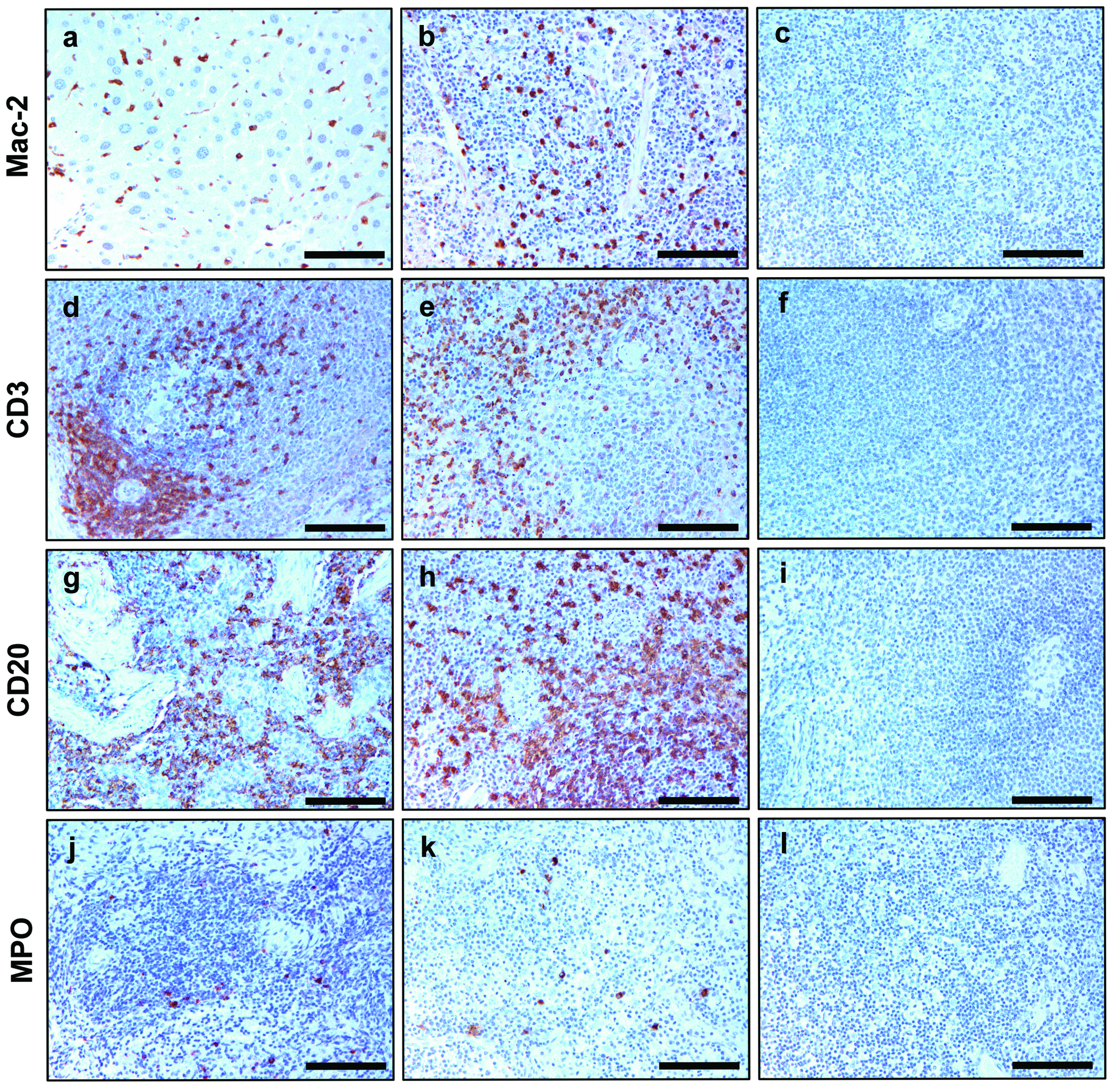

Supplement: Supplementary file 4 — Supplementary Figure 2 [file 41392_2022_891_MOESM4_ESM.tif]
